# CRISPR‐Scan: Cas13a Mismatch Tolerance Enabled Ultra‐Multiplexed Detection of Same‐Site Indels in Acute Myeloid Leukemia

**DOI:** 10.1002/advs.78229

**Published:** 2026-10-14

**Authors:** Linlin Bai, Bei Wang, Mingmin Shi, Dianwei Liu, Huimin Ji, Ting Wang, Ziwei Wang, Kaiming Guo, Xinyang Dou, Chunyan Qian, Fang Zhang, Yongming Wang, Hongju Mao, Rui Wang

**Affiliations:** ^1^ Center for Medical Research and Innovation Department of Laboratory Medicine Shanghai Pudong Hospital Human Phenome Institute State Key Laboratory of Genetics and Development of Complex Phenotypes School of Life Sciences Fudan University Shanghai China; ^2^ State Key Laboratory of Digital Medical Engineering Department of Otolaryngology Head and Neck Surgery School of Medicine Advanced Institute for Life and Health Zhongda Hospital Jiangsu Province High‐Tech Key Laboratory for Bio‐Medical Research Southeast University Nanjing China; ^3^ Department of Hematology Peking University Shenzhen Hospital Shenzhen Peking University‐The Hong Kong University of Science and Technology Medical Center Shenzhen China; ^4^ Department of Gastroenterology Shanghai Institute of Pancreatic Diseases National Key Laboratory of Immunity and Inflammation Changhai Hospital Naval Medical University Shanghai China; ^5^ Clinical Laboratory Linping Campus The Second Affiliated Hospital of Zhejiang University School of Medicine Hangzhou China; ^6^ College of Biological Science and Engineering Fuzhou University Fuzhou China; ^7^ Research Institute for Microbiology and Department of Food Science and Nutrition The Hong Kong Polytechnic University Kowloon Hong Kong China; ^8^ International Human Phenome Institutes Shanghai China

**Keywords:** acute myeloid leukemia (AML), CRISPR/Cas13, CRISPR‐Scan, mismatch tolerance, *NPM1* insertion, same‐locus indels

## Abstract

Detection of gene mutations related to acute myeloid leukemia (AML) is crucial for its early diagnosis, personalized treatment, and recurrence monitoring. However, accurate detection of diverse mutations occurring at the same locus remains technically challenging. The CRISPR/Cas system, with its unique programmable nucleic acid recognition capabilities, provides a promising solution. Here, we elucidated that CRISPR/LwaCas13a is tolerant to consecutive substitutions but highly sensitive to insertions or deletions (indels) at specific positions of the spacer sequence, enabling rational CRISPR RNA (crRNA) design for multiplex detection of same‐site indels. Guided by these principles, we achieved effective detection of ultra‐multiplex *nucleophosmin 1* (*NPM1*) c.863_864 4‐bp insertions with a single crRNA, while maintaining specificity against the wild‐type transcripts. We further established a one‐pot CRISPR system, CRISPR‐Scan, by integrating recombinase polymerase amplification with LwaCas13a cleavage. CRISPR‐Scan enabled detection of mutations in backgrounds as low as 0.01%. In clinical validation, it identified *NPM1* mutations in 43 of 120 AML patients, consistent with next‐generation sequencing (NGS). Continuous monitoring of four AML patients indicated early recurrence with higher sensitivity than first‐generation sequencing (FGS) and full concordance with NGS. A microfluidic chip and portable device further facilitate accurate AML mutation detection. This platform offers an efficient and convenient approach for early AML diagnosis and measurable residual disease (MRD) monitoring.

## Graphical abstract

Accurately detecting diverse same‐locus insertions or deletions (indels) remains challenging. This study reveals the mismatch‐tolerance profile of LwaCas13a and establishes CRISPR‐Scan, which uses a single crRNA to detect multiple same‐locus indels. The one‐pot assay detects *nucleophosmin 1* (*NPM1*) mutant allele frequencies down to 0.01%, agrees with next‐generation sequencing in 120 samples, supports measurable residual disease monitoring, and integrates with portable microfluidics for point‐of‐care testing.

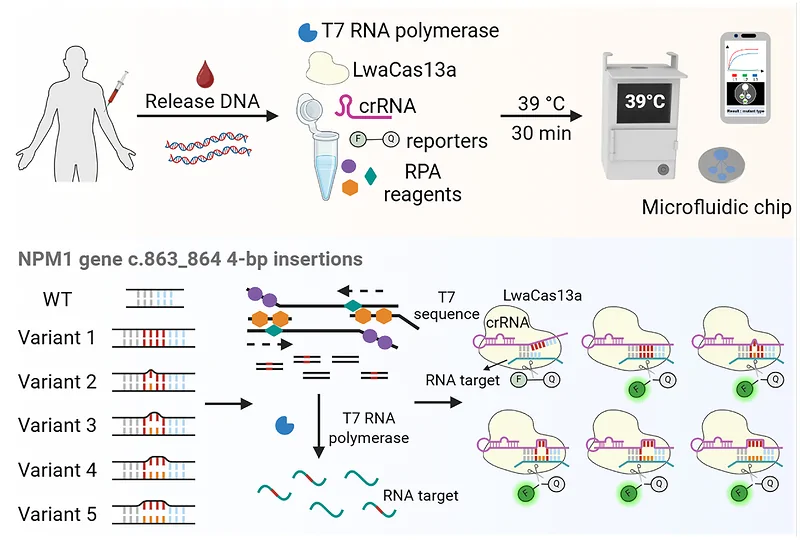

**Abbreviations**
AML: Acute myeloid leukemia
Cas: CRISPR‐associated
CRISPR: Clustered regularly interspaced short palindromic repeats
ddPCR: Droplet digital polymerase chain reaction
FGS: First‐generation sequencing
FISH: Fluorescence in situ hybridization
LOD: Limit of detection
MRD: Measurable residual disease
NGS: Next‐generation sequencing
*NPM1*
: Nucleophosmin 1
RPA: Recombinase polymerase amplification
sgRNA: Single guide RNA

## 1 Introduction

Advances in molecular genetics, genome sequencing technologies, and bioinformatics highlight the crucial role of genetic testing in early cancer detection, precise therapy selection, and disease monitoring [1, 2, 3]. Emerging research increasingly links gene mutations to tumorigenesis and progression, emphasizing the need for highly sensitive and accurate detection of gene mutations [4, 5, 6]. Insertions and deletions (indels) mutations are important types of genetic alterations that tend to cause frameshift, leading to abnormal protein function and promoting cancer development [7, 8, 9]. For instance, mutations in the *nucleophosmin 1* (*NPM1*) gene, typically involving a 4‐bp insertion at c.863_864 in exon 12, occur in approximately 30%–35% of adult acute myeloid leukemia (AML) cases and in approximately 50%–60% of cytogenetically normal AML cases [10, 11, 12, 13]. *NPM1* mutations result in aberrant cytoplasmic localization of mutant NPM1protein, leading to the loss of nucleolar localization and decreased stability of p14ARF, thereby weakening p14ARF‐mediated inhibition of MDM2 and disrupting the ARF‐MDM2‐p53 tumor suppressor pathway [14]. More than 100 types of *NPM1* 4‐bp insertion variants have been reported in AML [15]. *NPM1* c.863_864 4‐bp insertions show high stability during the clonal evolution of AML, making it a reliable molecular marker in AML diagnosis, and also the best molecular target for prognostic assessment with measurable residual disease (MRD) [16, 17]. Other typical examples include the KIT gene mutation p.W557_K558del, which is strongly associated with liver metastasis of gastrointestinal stromal tumors [18], and the oncogenic *BRAF* p.V487_T491del mutation, which is located in the β3‐αC loop [19]. Additionally, the *EGFR* p.E746_A750del mutation is a well‐established tyrosine kinase inhibitor (TKI)‐sensitive mutation in lung cancer [20, 21, 22]. Therefore, developing highly sensitive methods for indel detection is of substantial scientific significance and clinical utility.

The detection techniques for indel mutations mainly include Polymerase Chain Reaction (PCR) and its derivative methods (such as real‐time quantitative PCR, digital PCR) [23], fluorescence in situ hybridization technology (FISH) [24], first‐generation sequencing (FGS) [25], and next‐generation sequencing technology (NGS) [26]. However, PCR‐based methods detect only a limited subset of high‐prevalence variants within the mutational spectrum of a given gene. Even current multiplex PCR methods are unable to simultaneously detect dozens of mutation types at the same locus, thereby limiting the coverage of gene mutations at the same site. This limitation is primarily due to the challenges in probe design when multiple random mutations occur at the same position, making comprehensive detection difficult [27]. FISH can detect structural abnormalities of gene mutations, but it is mainly applicable to chromosomal‐level variations [28]. For detecting indels or substitutions, its sensitivity is not as good as PCR [29]. FGS can generally detect mutations with variant allele frequencies above 5% [25]. NGS can detect all types of gene mutations, including those undetectable by PCR, but its clinical application, especially for MRD monitoring, is limited due to the long turnaround time, typically more than two weeks, high cost, and the need for specialized technicians and complex data analysis [30, 31]. Therefore, it is still necessary to develop a sensitive, rapid, low‐cost, and convenient method to achieve comprehensive detection of gene mutations.

The clustered regularly interspaced short palindromic repeats (CRISPR)/CRISPR‐associated (Cas) system, as a revolutionary biotechnological tool, has demonstrated unparalleled potential in molecular diagnosis due to its ease of operation, high sensitivity, and specificity [32]. Jennifer Doudna's group established the CRISPR/Cas12a‐based DNA mutation detection system, DNA endonuclease‐targeted CRISPR trans reporter (DETECTR), which enables precise single‐nucleotide polymorphisms (SNPs) discrimination [33]. Feng Zhang's team developed the CRISPR/Cas13‐based specific high‐sensitivity enzymatic reporter unlocking (SHERLOCK) platform, achieving single‐molecule sensitivity for detecting circulating tumor DNA (ctDNA) [34]. However, existing CRISPR‐based detection systems mainly target predefined mutations and lack the capacity to detect diverse mutations at the same site simultaneously. Previous studies have shown that compared with Cas12 enzymes, Cas13 enzymes show greater mismatch tolerance, thereby demonstrating potential for random multiplex mutation detection [35]. In addition, *Leptotrichia wadei* Cas13a (LwaCas13a) is unrestricted by protospacer adjacent motif (PAM) sequences, enriching its flexibility. Upon recognition of the target RNA, Cas13 is activated, triggering collateral cleavage of a fluorophore‐quencher‐tagged RNA reporter, which releases the fluorescent signal for detection [34]. However, the mismatch tolerance rule of Cas13 is not well understood. More work in developing prediction tools that can design CRISPR RNAs (crRNAs) with desired substitution mismatch tolerance, and new crRNA design strategies that can distinguish random indels at the same site from the wild‐type (WT) sequence, is required to overcome this challenge. Further advances in molecular diagnostics demand an in‐depth understanding of the mismatch tolerance mechanism of Cas13, which can guide the rational design of highly specific Cas13‐based RNA detection strategies.

In this study, we developed a simultaneous Cas13a‐mediated amplification‐enhanced nucleic acid profiling (CRISPR‐Scan) system to systematically investigate how mismatch location and quantity affect the specificity of LwaCas13a, and to elucidate its mismatch tolerance rule. Applying these design principles, we engineered programmable crRNAs capable of performing both “exact match” and “approximate pattern match” of targets, enabling a single crRNA to simultaneously detect multiple same‐site indels while preventing detection of the wild‐type sequence. Using AML detection as an example, we rationally designed a crRNA to cover all 256 possible 4‐bp insertion combinations at the *NPM1* c.863_864 locus. We further coupled LwaCas13a cleavage with recombinase polymerase amplification (RPA) in a one‐pot reaction, achieving attomolar (aM) sensitivity. We applied the CRISPR‐Scan system to detect *NPM1* insertion mutations in 120 AML patients and to monitor MRD recurrence in a subset of cases during treatment follow‐up, demonstrating a detection limit of 0.01%. Moreover, to make this method more convenient for point‐of‐care testing (POCT), we developed a smart integrated microfluidic portable device, establishing an efficient, sensitive, and convenient tool for rapid detection of ultra‐multiplex same‐site indels in clinical diagnostics.

## 2 Materials and Methods

### 2.1 Materials and Reagents

The TwistAmp Basic Kit (Cat. No. TABAS03KIT) was obtained from TwistDx (Cambridge, UK). LwaCas13a (Cat. No. E0803H1) was obtained from Hylegen Bioscience (Shanghai, China). Recombinant RNase Inhibitor (Cat. No. 2313A) and Taq DNA Polymerase (Cat. No. R007A) were purchased from Takara Bio (Dalian, China). T7 RNA Polymerase (Cat. No. M0251L) was obtained from New England Biolabs (MA, USA). The Midi Purification Kit (Cat. No. DP209) and Genomic DNA extraction Kit (Cat. No. DP304) were obtained from Tiangen Biotech (Beijing, China). KOD FX DNA Polymerase (Cat. No. KFX‐101) was obtained from TOYOBO (Shanghai, China). The 2× BioZues Taq PCR Master Mix (Cat. No. SM0111‐005) was purchased from Bioligo Biotech (Shanghai, China). The HiPure RNA Pure Micro Kit (Cat. No. R2144‐03) was obtained from Magen Biotech (Guangzhou, China). All oligonucleotides were synthesized and modified by Sangon Biotech (Shanghai, China) (Tables S1–S11).

### 2.2 Ethics Statement

All clinical samples were provided by the First People's Hospital of Linping District, Shanghai Changhai Hospital, and Peking University Shenzhen Hospital. The study protocols were reviewed and approved by the respective institutional ethics committees of the participating hospitals (approval numbers: LPFH‐EC‐2025‐258, CHEC2022‐247, and PKUSZH‐EC‐2026‐209, respectively). Written informed consent was obtained from all participants.

### 2.3 LwaCas13a‐Based Cleavage

LwaCas13a‐based cleavage system included 4 U Recombinant RNase Inhibitor, 50 nm LwaCas13a, 25 nm crRNA, 120 nm single‐stranded RNA (ssRNA) reporter, 1×Reaction buffer, RNA template, and RNase‐free water up to 20 µL. After incubation at 37°C for 30 min, fluorescent results inside the tube were directly observed with the naked eye under blue light.

### 2.4 CRISPR‐Scan System

One tube of lyophilized RPA enzyme mix was reconstituted with 29.5 µL of rehydration buffer and mixed thoroughly. A 14.7 µL aliquot of the reconstituted mixture was used for each 30 µL CRISPR‐Scan reaction. NTPs at a total concentration of 4 mm (1 mm each ATP, CTP, GTP, and UTP), 75 U T7 RNA Polymerase, 480 nm each of forward and reverse primers, 40 U Recombinant RNase Inhibitor, 150 nm LwaCas13a, 300 nm crRNA, 500 nm ssRNA reporter, 80 ng template DNA, and DEPC‐treated water to a final volume of 28.5 µL. Then 14 mm MgOAc was added to adjust the total volume to 30 µL and start the reaction. The reaction was incubated at 39°C for 30 min, during which fluorescence signals were recorded at 1‐min intervals using a real‐time PCR instrument (Jingle, Hangzhou, China).

### 2.5 Statistical Analysis

Statistical analyses were performed using GraphPad Prism 8.0 (GraphPad Software, CA, USA) and Origin 2024 (OriginLab, Northampton, MA, USA). Data normalization was performed as indicated in the corresponding figure legends, and no data points were excluded as outliers. Unless otherwise specified, data were presented as the mean ± standard deviation (s.d.). Statistical significance was assessed using an unpaired two‐tailed Student's *t*‐test for two independent groups. The effects of mismatch location and mismatch number in the profiling experiments were assessed by two‐way analysis of variance (ANOVA) without post‐hoc pairwise comparisons. Weighted linear regression was performed using Origin 2024 to assess the relationship between endpoint fluorescence intensity and log_10_‐transformed variant allele frequency (VAF, %), with weights set to the inverse square of the s.d. at each concentration. A *p* value < 0.05 was considered statistically significant. The exact sample size (*n*) is specified in each figure legend. Where *n* = 3, the data were obtained from three separately prepared reactions, which represent technical replicates.

## 3 Results

### 3.1 The Workflow of CRISPR‐Scan

Currently, over 100 types of 4‐bp insertions have been discovered in the *NPM1* gene of AML patients. To address the challenge of detecting multiple same‐site indels, we developed the CRISPR‐Scan system, which systematically investigated the effects of mismatch position and quantity on the specificity of LwaCas13a and obtained its mismatch tolerance profile. Based on this profile, we rationally designed crRNAs capable of recognizing multiple variants with a single crRNA (Figure S1). In the workflow of the CRISPR‐Scan detection system, genomic DNA from patients was directly added to the one‐pot CRISPR detection system, and then real‐time fluorescence was monitored and reported by the portable homemade microfluidic integrated device (Figure 1a). Specifically, the reaction system includes T7 RNA polymerase, RPA reagents, a pair of primers, fluorophore‐quencher‐tagged RNA reporter, LwaCas13a, and a single crRNA. Variants of the *NPM1* c.863_864 4‐bp insertion, differing by only a few bases, can be recognized by the LwaCas13a/crRNA ribonucleoprotein (RNP) complex, which activates collateral ssRNA cleavage and emits strong fluorescence signals. In contrast, the wild‐type sequence cannot induce fluorescence due to its substantial difference from the crRNA, thus ensuring the specificity of the CRISPR‐Scan detection system (Figure 1b). Moreover, LwaCas13a lacks *cis*‐cleavage activity against target DNA, thus avoiding target depletion and enabling high reaction efficiency and sensitivity.

**FIGURE 1:**
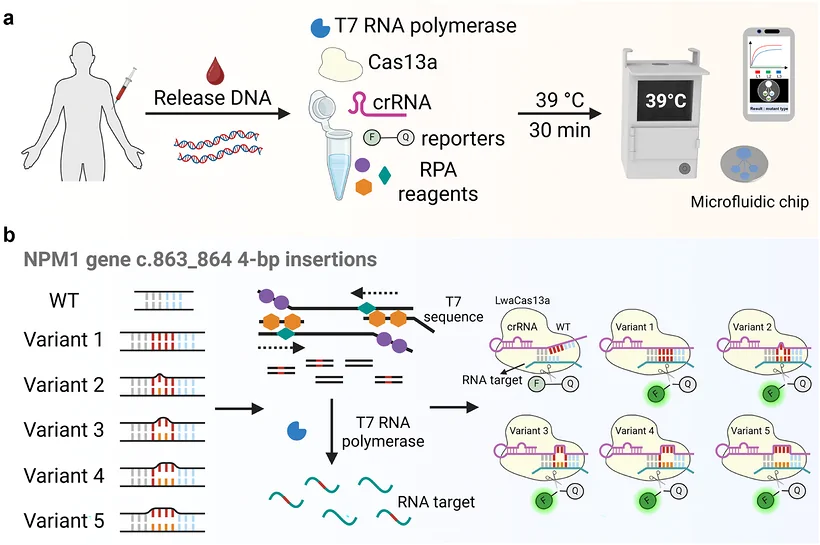
The reaction principle and workflow of the CRISPR‐Scan system. (a) Workflow of the CRISPR‐Scan detection system. Genomic DNA was extracted from patient blood samples, and 1 µL of genomic DNA was directly added to the CRISPR‐Scan reaction mixture, which contained T7 RNA polymerase, RPA reagents, a pair of primers, RNA reporter, LwaCas13a, and a single crRNA. The one‐pot reaction was performed at 39°C for 30 min inside the homemade microfluidic device. Real‐time fluorescence curves and visual detection results were recorded with a smartphone, and detection results were output automatically. (b) For CRISPR‐Scan detection, the mutant or wild‐type (WT) template was amplified using primers containing a T7 promoter sequence by RPA, followed by T7 transcription to produce RNA products. LwaCas13a/crRNA recognized RNA targets with 1–4 bp mismatches, activated its *trans*‐cleavage activity, and induced cleavage of the RNA reporter, which produces fluorescence signals. However, the WT RNA contains a large number of base mismatches, making it difficult for LwaCas13a/crRNA to recognize, and therefore no fluorescence signals were generated. LwaCas13a/crRNA can achieve the distinction between WT and *NPM1* gene c.863_864 4‐bp insertions, while also covering the detection of 4‐bp random insertion mutations. Created within BioRender.com. Bai, L. (2026) https://BioRender.com/12nctes.

### 3.2 Profiling Mismatch Sensitivity in CRISPR/Cas13a‐Mediated Recognition

To systematically investigate the mismatch tolerance of LwaCas13a in response to target RNA, we analyzed the *trans*‐cleavage activity of a panel of 21 RNA templates, each designed with 2–4 consecutive mismatches, spanning the entire target region complementary to the spacer sequence, represented as LnQm (Figure 2a,b, Table S1). Ln is the digital position identifier of the mismatch within the sequence, and Qm represents the number of mismatched bases. The 28 bp target region was divided into seven 4‐bp fragments (LR1 to LR7), and within each segment, 2 to 4 consecutive mismatches were introduced. In vitro LwaCas13a cleavage assay demonstrated that LwaCas13a exhibits position‐ and mismatch number‐dependent specificity (Figure 2c,d). 2 and 3 bp consecutive mismatches were generally tolerated by LwaCas13a, showing approximately 80%–100% of the cleavage activity, except for 3 bp consecutive mismatches in the LR5 region, which resulted in a substantial decrease to approximately 30%. 4 bp consecutive mismatches in LR5 region were poorly tolerated, reducing the cleavage activity to around 14%. In contrast, tolerance to 4‐bp consecutive mismatches generally increased with increasing distance from LR5, with LR1‐LR3 showing activity comparable to the perfectly matched target, around 70% activity observed in LR4, and 50% in LR6‐LR7. Therefore, when random mutations are located in the LR1‐LR4 region, LwaCas13a could better cover the detection of random mutations.

**FIGURE 2:**
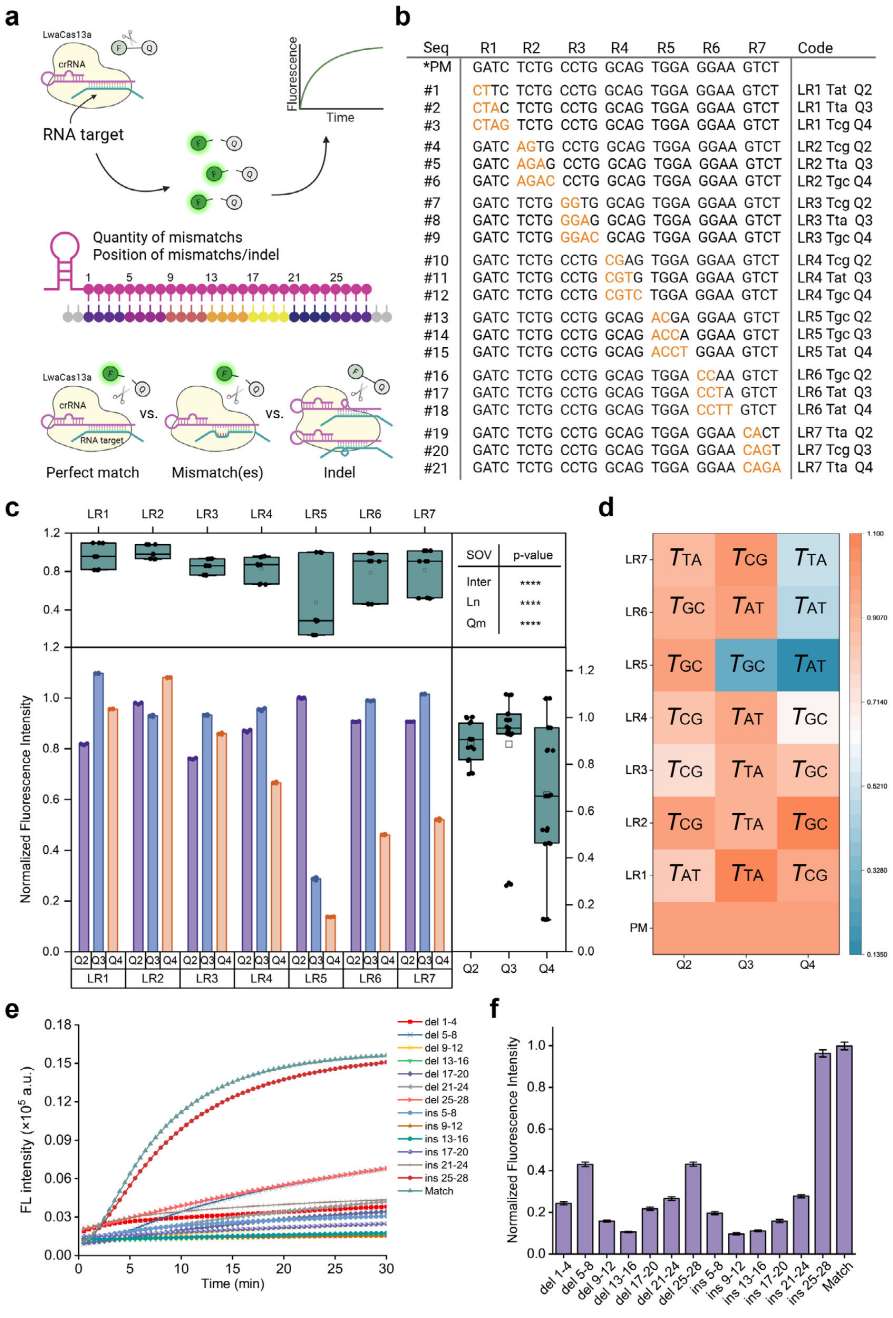
LwaCas13a exhibits different specificity to substitutions and indels in a position‐ and base‐count‐dependent manner. (a) Schematic diagram of the LwaCas13a RNA detection method, which utilized the *trans*‐ssRNA cleavage activity of the enzyme to cleave the quenched fluorescent RNA reporter. The target RNA was divided into 7 regions. By introducing different numbers of mismatch bases or indels in different regions, the differences in the *trans*‐cleavage activity of LwaCas13a were explored, with a perfect‐match (PM) RNA template as reference. (b) Mismatch patterns within the target RNA. Mismatches are highlighted in orange. PM represents a perfect match. The displayed sequence is from *NPM1*. (c) Normalized endpoint fluorescence signals of the LwaCas13a *trans*‐cleavage assay with varying mismatch positions and quantities. Data grouped by mismatch location (Ln) and mismatch number (Qm) are illustrated as box plots. Center lines indicate medians, boxes indicate interquartile ranges, whiskers indicate lower and upper adjacent values, black points indicate individual observations, and open squares indicate means. The upper‐right inset shows the results of two‐way ANOVA. Data shown are means ± s.d. (*n* = 3 technical replicates). SOV: source of variation; ^****^ *p*‐value < 0.0001. (d) Heatmap of normalized fluorescence signals of the LwaCas13a *trans*‐cleavage assay in c, displayed with base substitution (Txy). Squares are colored by normalized cleavage activity, according to the color bars on the right, with orange and blue indicating higher and lower cleavage activity, respectively. (e) Fluorescence curves of the LwaCas13a cleavage assay with a 4‐bp indel at different locations. (f) Normalized endpoint fluorescence signal at 30 min. Data shown are means ± s.d. (*n* = 3 technical replicates).

Furthermore, we explored the indel mismatch tolerance of LwaCas13a. We introduced insertion or deletion mismatches into the target RNA at LR1‐LR7 regions. Results showed that all deletion mismatches resulted in obvious reductions in cleavage activity, with deletions in LR2 and LR7 reducing activity to around 50%, and those in LR1, LR5, and LR6 decreasing it to 20%‐25%. Notably, deletions located in the LR3 and LR4 regions exhibited the strongest inhibitory effect on LwaCas13a *trans*‐cleavage activity, lowering the residual activity to nearly 10%. Insertion mismatches in LR2‐LR6 also showed a substantial reduction in activity, whereas the insertion in LR7 had no observed effect (Figure 2e,f). These findings highlight the feasibility of detecting ultra‐multiplex same‐site insertion mutations using rationally designed crRNAs by leveraging the substitution mismatch tolerance and indel mismatch intolerance of LwaCas13a.

To assess the generalizability of this design principle, we performed corresponding mismatch profiling in two additional sequence contexts, *Pseudomonas aeruginosa* (PA) and *BRAF*. For each target, we analyzed 21 RNA templates containing 2–4 consecutive substitution mismatches across LR1‐LR7, together with templates carrying 4‐bp insertions or deletions in the same regions. In the *PA* context, LR4‐LR5 retained approximately 75%–100% activity toward substitution mismatches, whereas indel mismatches reduced activity to approximately 13%–29% (Figures S2 and S3, Table S2). In the *BRAF* context, LR4 retained approximately 50%–100% activity toward substitution mismatches, whereas indel mismatches reduced activity to approximately 10% (Figures S4 and S5, Table S3). Although the precise positional mismatch‐tolerance patterns varied among *NPM1*, PA, and *BRAF*, each sequence contained regions that tolerated consecutive substitutions while remaining sensitive to indels. These results indicate that spacer regions with high substitution tolerance and high indel sensitivity are favorable for designing a single crRNA to detect multiple same‐site indel variants. Moreover, the optimal targeting region is therefore sequence‐context dependent and should be experimentally determined for each locus.

### 3.3 Detection of Ultra‐Multiplex Insertion Mutations in *NPM1* Gene With LwaCas13a Cleavage Assay

To verify the capability of LwaCas13a to detect 4‐bp random insertions using a single crRNA, we designed and screened crRNAs targeting the c.863_864 4‐bp insertions in the *NPM1* gene. As reported, 11 insertion types account for over 97% of the *NPM1* mutation cases in AML, with insTCTG (76.4%), insCATG (9.2%), and insCCTG (7.2%) representing the most common types. Given the minimal nucleotide difference between insCCTG and the other 10 highest insertion mutations, we designed crRNA (*NPM1*‐crRNA1∼10) that complements the insCCTG sequence, rather than the most common insTCTG sequence, so as to obtain stronger fluorescent signals in a variety of high‐frequency insertion mutation detection (Figure 3a and Figure S6, Tables S4 and S5). Since LwaCas13a is not restricted by the PAM site, we designed 5 crRNA sequences targeting each of the sense and antisense strands of the template, respectively. To identify the most specific crRNA, we measured the endpoint fluorescence ratio of insCCTG to wild‐type DNA for the 10 crRNAs. Among them, crRNA 9–12R showed strong specificity and the highest fluorescence signal for insCCTG detection (Figure 3b). To further evaluate whether this crRNA could detect other *NPM1* insertions beyond insCCTG, we synthesized ten oligonucleotide fragments for each mismatch number (1–4 bp) relative to insCCTG. These fragments were subjected to PCR amplification, in vitro transcription, and purification, and the resulting RNA products were used for in vitro LwaCas13a cleavage assay (Figure 3c, Table S6). Results indicate that the LwaCas13a/crRNA 9–12R complex exhibits strong tolerance toward 1–4 bp mutations (Figure 3d), further demonstrating the CRISPR‐Scan system's robust detection potential for over 100 identified *NPM1* c.863_864 4‐bp insertions. Finally, using the LwaCas13a/crRNA 9–12R, we successfully detected the 11 most common *NPM1* gene c.863_864 4‐bp insertions in AML cases. All of the tested insertion variants produced robust fluorescence signals, with no significant cross‐reactivity with wild‐type DNA (Figure 3e and Figure S7, Table S7).

**FIGURE 3:**
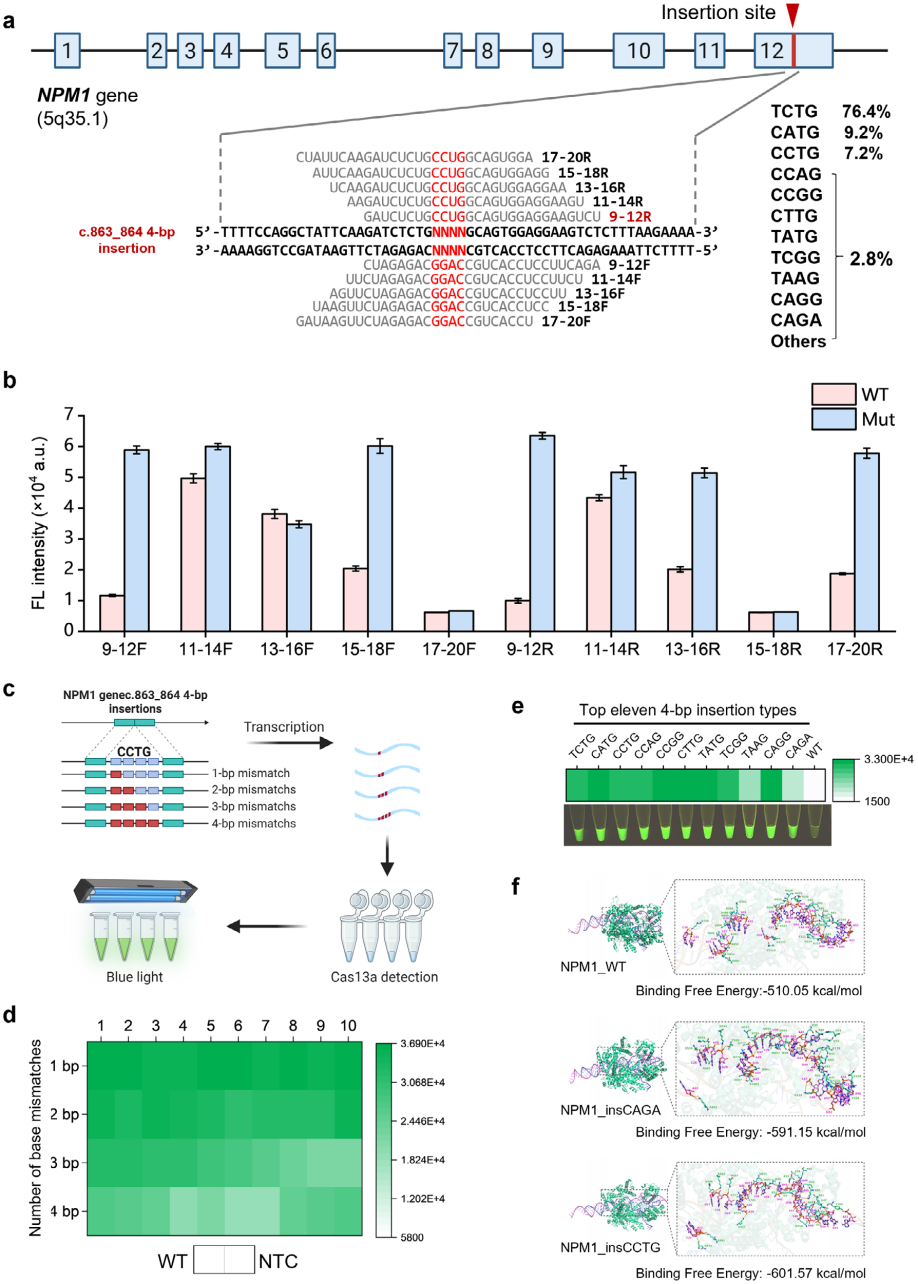
Design and selection of crRNA enable the detection of multiple same‐site insertion mutations of the *NPM1* gene. (a) The genomic location of the 4‐bp insertion at position c.863_864 in the *NPM1* gene is marked with a red triangle. Representative mutant rates are listed on the right. The sequences of target DNA and 10 designed crRNAs, complementary to either the sense or antisense strand, are shown on the bottom. The inserted bases are marked in red. (b) End‐point fluorescence values of the LwaCas13a *trans*‐cleavage assay with 10 designed crRNAs. WT, wild type; Mut, mutant (insCCTG). Data shown are means ± s.d. (*n* = 3 technical replicates). (c) Utilize crRNA 9–12R to identify templates that have 1–4 base mismatches compared to the *NPM1* insCCTG template. Created in BioRender. Bai, L. (2026) https://BioRender.com/bv0q2fi. (d) Heatmap of endpoint fluorescence values of LwaCas13a/crRNA *trans*‐cleavage assay when the templates contain 1–4 mismatches relative to the crRNA. NTC, no‐template control. (e) Representative endpoint fluorescence intensity and visualization results of CRISPR‐Scan detection for the top 11 highest frequency types of c.863_864 4‐bp insertions with LwaCas13a/crRNA 9–12R. (f) The schematic diagram of the binding of LwaCas13a/crRNA 9–12R with WT, insCCTG, and insCAGA of *NPM1* gene, as well as the binding free energy.

To understand the theoretical binding of LwaCas13a/crRNA 9–12R to the *NPM1* gene c.863_864 4‐bp insertion template, we employed AlphaFold3 for de novo modeling to predict the structure of the ternary complex formed by LwaCas13a/crRNA 9–12R and the template RNA. A 100 ns molecular dynamics simulation was performed on the complex to further investigate the binding site and affinity of the LwaCas13a/crRNA 9–12R and template RNA complex. We selected three subjects: *NPM1*_WT, *NPM1*_insCCTG, and *NPM1*_insCAGA, with *NPM1*_insCCTG completely pairing with crRNA and *NPM1*_insCAGA having three base mismatches with crRNA. Through kinetic simulations, it was found that in binding free energy analysis, the LwaCas13a/crRNA 9–12R exhibited the lowest binding free energy of −601.57 kcal/mol with the *NPM1*_insCCTG target, and a binding free energy of −591.15 kcal/mol with the *NPM1*_insCAGA target, which is comparable to the *NPM1*_insCCTG target. The highest binding free energy observed for the *NPM1*_WT target was −510.05 kcal/mol (Figure 3f). In hydrogen bond analysis, the LwaCas13a/crRNA 9–12R formed up to 119 hydrogen bonds with the *NPM1*_insCCTG target and 118 bonds with the *NPM1*_insCAGA target. This is comparable to the *NPM1*_insCCTG target, while the fewest hydrogen bonds (112 bonds) were observed with the *NPM1*‐WT target. In the interaction analysis, LwaCas13a/crRNA 9–12R exhibited the highest number of interactions with the *NPM1*_insCCTG target (258 interactions), followed by the *NPM1*_insCAGA target (233 interactions), and the lowest number of interactions with the *NPM1*_WT target (213 interactions). The data above indicate that the LwaCas13a/crRNA 9–12R complex exhibits the strongest binding affinity for the *NPM1*_insCCTG target, followed by the *NPM1*_insCAGA target with binding strength comparable to that of *NPM1*_insCCTG. It demonstrates the weakest binding affinity for the *NPM1*_WT target (Figures S8 and S9). Overall, we confirmed that LwaCas13a owns the ability to detect multiple insertions using a single crRNA without PAM constraints.

### 3.4 Establishment and Optimization of CRISPR‐Scan Detection System

To achieve rapid, convenient detection, we combined LwaCas13a/crRNA cleavage with RPA amplification, establishing a one‐pot CRISPR‐Scan detection system. We optimized the initial system by varying multiple parameters, including the efficiency of the RPA amplification, T7 transcription, and LwaCas13a‐mediated cleavage processes. For optimization of the RPA reaction system, we used 1e4 copies of the *NPM1*‐expressing plasmid as the template and screened 25 primer pairs, consisting of 5 forward primers (F1‐F5) and 5 reverse primers (R1‐R5). The primer pair F3/R4, which showed the highest amplification efficiency, was selected for subsequent experiments (Figure 4a, Table S8). We also investigated the effect of temperature and found that 39°C yielded the highest reaction efficiency (Figure 4b,c). Next, we optimized NTP buffer concentration in the transcription system and found that 4 mm NTP buffer concentration induced the highest reaction efficiency (Figure 4d,e). We also optimized the concentration of T7 RNA polymerase, and 75 U of T7 RNA polymerase demonstrated the highest reaction efficiency (Figure 4f,g). Furthermore, we optimized the components of the LwaCas13a cleavage system. The reaction buffer of LwaCas13a exhibited an inhibitory effect on the overall one‐pot reaction and was therefore removed (Figure 4h,i). Overall, the experimental results demonstrated that 150 nm LwaCas13a, 300 nm crRNA 9–12R, and 500 nm reporter yielded the strongest specificity and relatively high reaction efficiency (Figure 4j–o).

**FIGURE 4:**
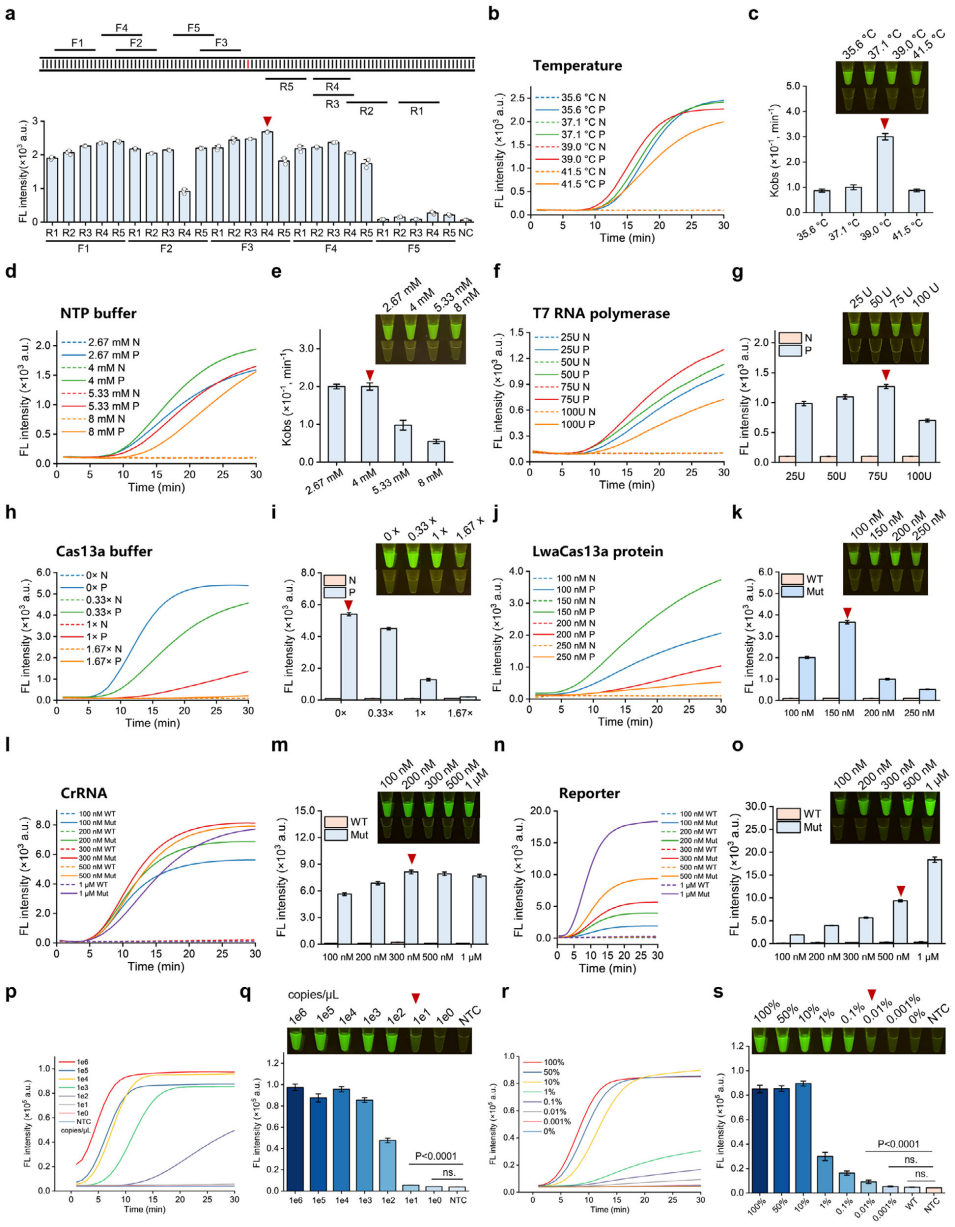
CRISPR‐Scan system set up and optimization. (a) The figure above shows the locations of the RPA primers designed for the *NPM1* target. The 4‐bp insertion site is indicated in red. Below are the amplification efficiency of 25 pairs of RPA primers. Plasmids containing the *NPM1* c.863_864insCCTG mutations, with 1e4 copies, were subjected to RPA amplification at 39°C for 20 min. Then, 0.5 µL of the amplification product was used for the LwaCas13a/crRNA 9–12R cleavage experiment at 37°C for 20 min, The best‐performing primer pair F3/R4 is indicated by a red triangle. NC, no‐template control. Data shown are means ± s.d. (*n* = 3 technical replicates). (b–o) Representative fluorescence curves and visualization results measured using the integrated one‐pot CRISPR‐Scan system under varying conditions, including: (b,c), reaction temperature; (d,e), NTP buffer concentration; (f,g), T7 RNA Polymerase concentration; (h,i), LwaCas13a buffer concentration; (j,k), LwaCas13a enzyme concentration; (l,m), crRNA concentration; (n,o), reporter concentration. N: Negative control; P: Positive. (p,q) Sensitivity evaluation of the CRISPR‐Scan system with 10‐fold serial dilutions of plasmids containing *NPM1* c.863_864 insCCTG mutations as templates. Fluorescence curve, endpoint fluorescence value, and visualization results were recorded. NTC: No template control. (r,s) Sensitivity evaluation of the CRISPR‐Scan system with a gradient of *NPM1* c.863_864insCCTG mutant plasmids mixed with WT plasmids. Fluorescence curve, endpoint fluorescence value, and visualization results were recorded. WT, wild‐type; Mut, mutant (insCCTG); NTC, no‐template control. (p–s), Data shown are means ± s.d. (*n* = 3 technical replicates). The indicated groups were compared with NTC. Statistical significance was assessed using unpaired two‐tailed Student's *t*‐tests. A *p*‐value < 0.05 was considered statistically significant; ns, not significant.

To further determine the limit of detection (LOD) of the optimized CRISPR‐Scan system, we first applied it to detect a 10‐fold serial dilution of the *NPM1*‐containing plasmid spanning six orders of magnitude. Results showed that it could detect as few as 10 copies per reaction, demonstrating its high sensitivity (Figure 4p,q). Furthermore, to investigate the specificity of the CRISPR‐Scan detection system for mutant‐doped sample determination, we prepared plasmid templates by mixing mutant and wild‐type plasmids at *NPM1* mutation frequencies ranging from 100% to 0.001%, and performed detection with 1e6 template copies per reaction. Results showed that the CRISPR‐Scan method could detect the 0.01% insCCTG mutation from high background interference, demonstrating its ultrahigh sensitivity and specificity (Figure 4r,s). We further employed the CRISPR‐Scan method to detect both insTCTG and insCATG mutations under high background interference. This achieved a detection sensitivity of 0.01% in plasmid DNA, with no cross‐reactivity observed in wild‐type templates (Figure S10). Moreover, additional analytical sensitivity testing of the relatively low‐signal insTAAG and insCAGA variants showed that both remained detectable at a mutant allele frequency of 0.01% under the optimized conditions (Figure S11). These results demonstrate the comprehensive coverage capability and high specificity of the CRISPR‐Scan system.

To ensure effective detection of genomic DNA carrying different *NPM1* mutation types, we conducted tests on the blood genomic DNA of three AML patients to determine the appropriate input template amount for CRISPR‐Scan detection. Different amounts of input genomic DNA were compared. Results showed that with input template added, the fluorescence signal demonstrated a trend of initially increased and subsequently decreased, and the range of 50–100 ng was determined as the appropriate range for template input (Figure 5a and Figure S12). By mixing genomic DNA from mutant samples with wild‐type genomic DNA from healthy individuals, we prepared samples with mutant frequencies ranging from 32.9% to 0.001% and from 20.6% to 0.001%, respectively. With 80 ng of genomic DNA as input, the CRISPR‐Scan detection system could detect as low as 0.01% mutations for both insertion types (Figure S13a,b,d,e). Notably, a single *NPM1*‐crRNA 9–12R sequence, designed to be complementary to the insCCTG variant, was sufficient to detect all the above insertion types, with no cross‐reactive signal observed from the wild‐type templates. These findings demonstrated the comprehensive coverage and high specificity of the CRISPR‐Scan system as well as its potential for clinical sample detection.

**FIGURE 5:**
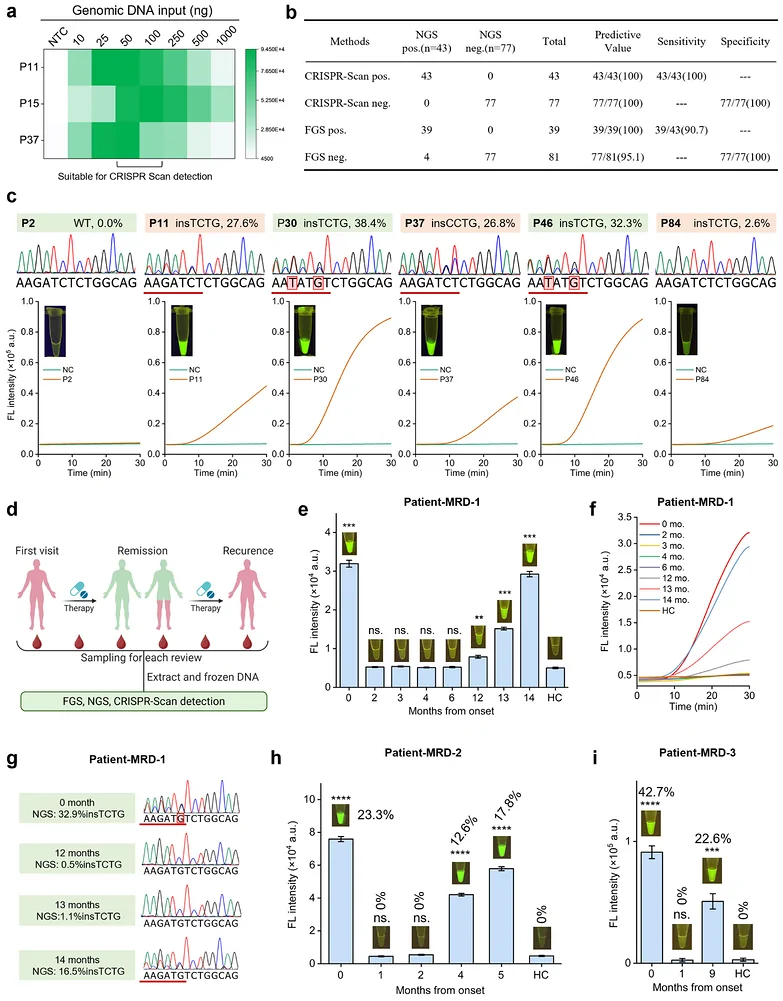
Detection of clinical samples with the CRISPR‐Scan system. (a) Endpoint fluorescence signals measured at 30 min for three AML patients with varying genomic DNA inputs. (b) Sensitivity and specificity of the CRISPR‐Scan method across 120 AML samples compared with NGS and FGS methods. (c) Representative NGS (top), FGS (middle), and CRISPR‐Scan system (bottom) results for AML patients. NC, no‐template control. (d), Workflow for MRD monitoring of AML patients. Created in BioRender. Bai, L. (2026) https://BioRender.com/8cl0cv. (e) Endpoint fluorescence signal and representative visualization results measured using CRISPR‐Scan system for Patient‐MRD‐1. Time points are shown as months from onset. (f) Representative real‐time fluorescence curves measured using the CRISPR‐Scan system for Patient‐MRD‐1. (g) Sequencing results for Patient‐MRD‐1. (h,i) CRISPR‐Scan and NGS results for *NPM1* c.863_864 4‐bp insertion from Patient‐MRD‐2 and 3. Endpoint fluorescence signals and representative visualization results obtained using CRISPR‐Scan system, together with the corresponding VAFs detected by NGS, were presented for each sample. Time points are shown as months from onset. (e, h, i) Data shown are means ± s.d. (*n* = 3 technical replicates). The indicated groups were compared with the healthy control (HC) sample. Statistical significance was assessed using unpaired two‐tailed Student's *t*‐tests. ^*^ *p* < 0.05, ^**^ *p* < 0.01, ^***^ *p* < 0.001, ^****^ *p* < 0.0001; ns, not significant.

### 3.5 Evaluation of CRISPR‐Scan Method for Detecting *NPM1* Mutations in Clinical Samples

To evaluate the clinical applicability of the CRISPR‐Scan system, we examined the *NPM1* mutations in 120 AML patients (P1 to P120) (Figure 5b,c, Figures S14 and S15). The genomic DNA from the bone marrow samples of 120 AML patients was extracted and analyzed by NGS, FGS, and CRISPR‐Scan analysis (Table S9 and S10). In the CRISPR‐Scan assay, after 30 min incubation at 39°C, 43 out of 120 samples demonstrated distinguishable fluorescence signals, fully consistent with the NGS results. However, the FGS method failed to detect 4 positive samples with low mutation rates (P55, 3.2% insTCTG; P76, 1.4% insTCTG; P84, 2.6% insTCTG, P108, 3.2% insTCTG). These results indicate that the CRISPR‐Scan method demonstrated higher sensitivity than FGS (100% vs. 90.7%). These results further validated the adaptability of the CRISPR‐Scan system for detecting diverse types of *NPM1* c.863_864 4‐bp insertions.

Previous studies have demonstrated that *NPM1* mutations represent an ideal molecular target for monitoring MRD in leukemia [16]. The CRISPR‐Scan system was used to detect residual leukemia in patients with *NPM1*‐mutated AML. This study analyzed the frozen bone marrow DNA samples of 4 patients (Patients‐MRD‐1 to 4). The samples were collected at multiple clinical time nodes, including initial diagnosis, remission, remission maintenance period, and recurrent follow‐up stages. The samples were analyzed using FGS, NGS, and CRISPR‐Scan, and the results were compared (Figure 5d). CRISPR‐Scan analysis of Patient‐MRD‐1 showed strong fluorescent signals in the sample collected at disease onset, indicating *NPM1* mutation positivity at initial diagnosis (Figure 5e,f, Figure S16). This finding was validated by NGS, which revealed an insTCTG mutation rate of 32.9% (Figure 5g). Following multiple rounds of chemotherapy, fluorescence signals disappeared in samples collected in 2, 3, 4, and 6 months after onset, consistent with MRD negativity during complete remission. NGS confirmed a mutation rate of 0.0% at these time points. Subsequently, in the sample collected 12 months after onset, the CRISPR‐Scan reaction showed a faint but significant fluorescent signal. This was confirmed by NGS, which identified a 0.5% insTCTG mutation rate, indicating detectable MRD with a recurrence trend. At months 13 and 14 after onset, the fluorescence signal increased progressively, consistent with the corresponding NGS variant allele frequencies of 1.1% and 16.5%, respectively. In contrast, FGS remained negative at month 13, demonstrating that the CRISPR‐Scan system provides an earlier molecular indication of leukemia relapse than FGS. Similar to Patient MRD‐1, CRISPR‐Scan analysis for another three MRD patients also perfectly correlated with disease progression and NGS findings (Figure 5h,i, Figures S17–S19). Given CRISPR‐Scan method's advantages in sensitivity, speed, convenience, and cost‐effectiveness, this technology is expected to become a convenient tool for MRD detection, offering immeasurable value in routine follow‐up and efficacy assessment for cancer patients.

Additional clinical characteristics for three of the four patients, including bone marrow blast percentages at the available time points, are summarized in Table S11. To examine whether CRISPR‐Scan signal reflected mutation abundance, patient‐derived genomic DNA harbouring *NPM1* mutation was serially diluted with wild‐type genomic DNA to generate samples with nominal variant allele frequencies (VAFs). Weighted linear regression analysis showed a positive linear relationship between endpoint fluorescence intensity and log_10_[VAF (%)] (R^2^ > 0.98) (Figure S13c, f), supporting the potential use of CRISPR‐Scan for MRD monitoring.

### 3.6 CRISPR‐Scan System Coupled With Homemade Smart Portable Microfluidic Device for Multiplexing Potential

To further verify the versatility of the CRISPR‐Scan system, we examined indel detection for more cancer‐related genes. First, we examined the *KIT* gene p.W557_K558del mutation, including two common variations of c.1669_1674del and c.1670_1675del, which are associated with liver metastasis of gastrointestinal stromal tumors [18] (Figure 6a, Table S12). We selected and identified one crRNA that could detect both variants with high sensitivity and without cross‐interference with wild‐type. It is worth noting that although there was a base mismatch between c.1670_1675del mutation and crRNA, its fluorescence signal strength was comparable to that of c.1669_1674del mutation, which confirmed that the CRISPR‐Scan system could use a single crRNA to recognize a variety of deletion mutations. In addition, this study used the CRISPR‐Scan system to detect the indels of *BRAF* and *EGFR* genes. *BRAF* p.V487_T491del mutation is an oncogenic driver mutation located in the β3‐αC loop, with two key mutations shown in Figure 6b [19]. The *EGFR* p.E746_A750del mutation is a recognized *TKI*‐sensitive alteration in lung cancer, with three key mutations shown in Figure 6c [21]. Similar to its performance in *KIT* detection, the CRISPR‐Scan system demonstrated high sensitivity and specificity in detecting both the *BRAF* p.V487_T491del and *EGFR* p.E746_A750del variants. Furthermore, the results showed that a single crRNA could sensitively detect two or three variants with 1 and 2 bp mismatches, and there was no significant cross‐interference with the wild‐type. These successful applications further demonstrated the multi‐functionality and effectiveness of the CRISPR‐Scan system in detecting gene insertions and deletions.

**FIGURE 6:**
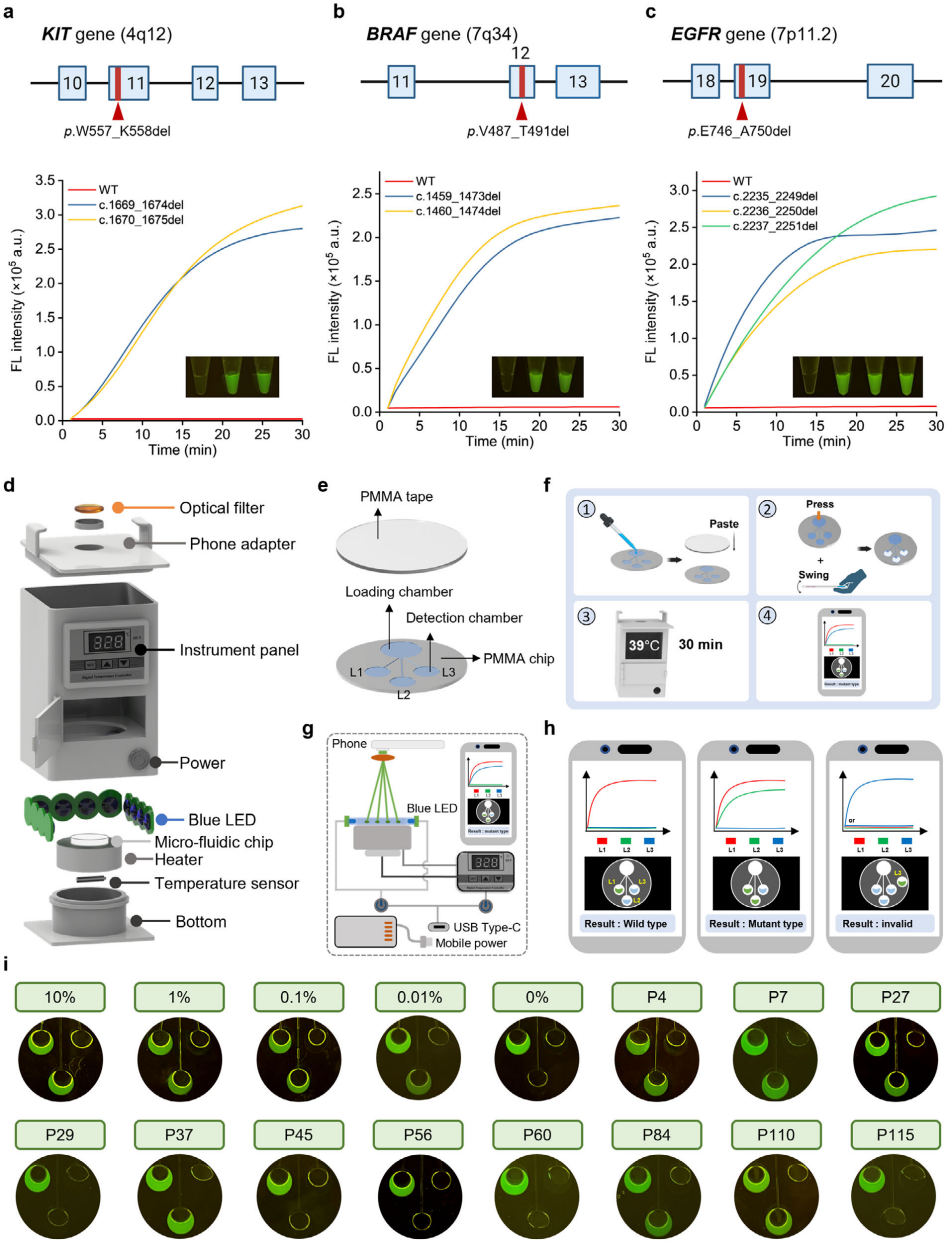
Expanded indel detection with CRISPR‐Scan system and integration with the homemade microfluidic device. (a–c) Schematic locations of the *KIT* p.W557_K558del, *BRAF* p.V487_T491del, and *EGFR* p.E746_A750del mutations, respectively (top), and the corresponding real‐time fluorescence curves and endpoint visualization images measured using CRISPR‐Scan system (bottom). Exons are represented by blue boxes, and deletion sites are marked with red triangles. (d) Exploded view schematic of the homemade integrated microfluidic device, comprising heating module, temperature sensor, blue‐light excitation module, and controller. Created with SolidWorks. (e) Schematic diagram of the microfluidic chip, comprising a sample loading chamber, three microchannels, and three detection chambers. Created with SolidWorks. (f) Microfluidic assay workflow. The CRISPR‐Scan reaction system containing *NPM1* primers, *GAPDH*‐target internal control (IC) primers, and *NPM1* crRNA 9–12R was added into the loading chamber. The left detection chamber (L1) contained sample genomic DNA and IC crRNA as the positive control, the right detection chamber (L3) contained IC crRNA and DEPC‐treated water as the negative control, and the middle detection chamber (L2) contained test sample genomic DNA. After sealing, the chip was pressed and gently agitated to distribute the reaction mixture among the three detection chambers, followed by incubation at 39°C for 30 min. Fluorescence signals could be directly observed by the naked eye and recorded with a smartphone. (g) The schematic of the portable device working principle. (h) Fluorescence collection and result analysis with a smartphone. Signals from L1, L2, and L3 are displayed as red, green, and blue curves, respectively. A wild‐type result is assigned when only L1 is positive, whereas a mutant result is assigned when L1 and L2 are positive, and L3 remains negative. All other signal patterns are classified as invalid. (i) Analytical sensitivity evaluation of the CRISPR‐Scan system in the microfluidic chip with 10%, 1%, 0.1%, and 0.01% mutant genomic DNA. Representative visualization results of the CRISPR‐Scan detection system for 11 AML clinical samples. The total amount of genomic DNA for all samples was 80 ng.

To improve the practicality of detection and eliminate false‐negative results in genetic testing caused by abnormal sample concentration, nucleic acid quality, or instrument malfunctions, we have developed a smart integrated microfluidic device (Figure 6d–g, Figure S20). The device features a rectangular appearance with a base housing a temperature sensor and a heating block. The temperature sensor provides real‐time feedback on the heating block's temperature, displayed on the dashboard. The microfluidic chip is placed on the heating block, surrounded by blue LED lights. The front of the enclosure includes a switch and a dashboard. The top of the device houses a phone holder equipped with a filter. During operation, the device is powered by a mobile power unit. Pressing the switch activates the blue LEDs. The target temperature is then set via the dashboard. After placing the microfluidic chip on the heating block, the mobile signal reading system is initiated for real‐time monitoring and curve output. Upon reaction completion, the final detection results are displayed at the bottom of the mobile screen automatically (Figure 6h). The microfluidic chip can perform three reactions simultaneously in a single loading step, including the negative control (NC) group, the Target group, and the internal control (IC) group. After the sample was loaded, the microfluidic chip was sealed with tape, effectively preventing aerosol contamination of amplified products and ensuring the reliability of the results. Figure 6e illustrated the structure of the PMMA microfluidic chip, which includes a sample loading chamber, three microchannels, and three detection chambers (Figure S21). When conducting the *NPM1* c.863_864 4‐bp insertion detection, the CRISPR‐Scan system containing *NPM1* crRNA 9–12R and primers was injected into the loading chamber. DNA template from sample and IC‐crRNA as positive control were pre‐placed in the left detection chamber (L1), while DEPC‐treated water and IC‐crRNA were placed in the right detection chamber as blank control (L3), and DNA template from sample was placed in the middle detection chamber for mutation detection (L2). After loading and sealing the sample with PMMA tape, the chip was gently pressed and swung to distribute the CRISPR‐Scan reaction mixture into the three detection chambers. The chip was then placed on the heating block inside the microfluidic device, and the one‐pot CRISPR reaction was performed at 39°C for 30 min (Figure 6f). The interpretation of the results depends on the fluorescence pattern. The presence of fluorescence in the left detection chamber (L1) indicates that the reaction system is working normally, thereby ruling out false negatives; the absence of a fluorescence signal in the right detection chamber (L3) indicates that there is no false positive contamination in the system; at this point, the intermediate detection chamber (L2) can be observed. The presence of fluorescence indicates the presence of a mutant target, and the absence of fluorescence indicates a wild‐type result. In other cases, the result is invalid (Figure 6h).

Furthermore, we evaluated CRISPR‐Scan integrated with the smart microfluidic device using mutant‐to‐wild‐type genomic DNA mixtures ranging from 10% to 0.01%, confirming a detection limit of 0.01%. Clinical validation was subsequently extended to a total of 51 patient samples, and the CRISPR‐Scan results showed high concordance with those obtained by NGS. Samples with high VAFs produced fluorescence signals that were readily visible to the naked eye, whereas samples with lower VAFs remained distinguishable from the negative controls despite their lower signal intensities (Figure 6i, Figure S22). These findings supported the robustness and the potential of the smart microfluidic device for point‐of‐care testing.

## 4 Discussion

CRISPR/Cas system has currently emerged as an effective tool for nucleic acid detection. Previous studies have reported that Cas13a exhibits poorer single‐base resolution and greater mismatch tolerance compared to Cas12a. Here, we systematically explored the principle of LwaCas13a/crRNA mismatch tolerance and elucidated the general considerations for any RNA‐detection application with LwaCas13a. We demonstrated that the binding affinity of the LwaCas13a/crRNA complex to the target RNA, as well as its RNase activity, is affected by the mismatches between the crRNA and target RNA. The LwaCas13a/crRNA complex can tolerate two consecutive mismatches. The toleration of three and four consecutive mismatches is position‐dependent. Increasing the number of mismatches and positioning them closer to positions 17–20 of the crRNA reduces the mismatch tolerance of LwaCas13a. For mismatches located at positions 1–16 of the crRNA, LwaCas13a exhibits higher mismatch tolerance. We also investigated the tolerance profile of LwaCas13a for 4‐bp indels in different regions of the crRNA. The results reveal that LwaCas13a shows low tolerance to 4‐bp deletions at all positions, as well as 4‐bp insertions within positions 1–24 of the crRNA. Moreover, we confirmed that MD simulations could be harnessed as a powerful tool for understanding the impact of mismatches on the allosteric coupling between spacer and target binding and Cas13 activation. Our work provides a platform for guiding the rational design of crRNAs for multiplex same‐site indel detection using the CRISPR/LwaCas13a system. The additional PA and *BRAF* profiling further shows that the key design principle of selecting a spacer region that is tolerant to consecutive substitutions but sensitive to indels is transferable across independent sequence contexts. However, the precise positional tolerance pattern and the optimal region varied among *NPM1*, PA, and *BRAF*. CRISPR‐Scan crRNAs should therefore be selected by locus‐specific empirical profiling rather than by assuming a universal spacer position.

We aimed to develop a CRISPR/Cas‐based molecular diagnostic system to address the challenge of detecting multiple variants at the same locus. Based on the principles of Cas13 mismatch tolerance, we revealed potential advantages for multi‐target recognition. *NPM1*‐mutated AML represents the most common subgroup of AML, accounting for around 30% of adult AML cases. Nearly all the *NPM1*‐mutated AML patients carry 4‐bp duplication/insertion mutations at position c.863_864 in exon 12. *NPM1*‐mutated AML is a favorable prognostic subtype of AML, with an overall survival (OS) rate of approximately 40% and the complete remission (CR) rate of around 80%. However, nearly 50% of patients eventually relapse. According to European Leukemia Net 2022 (ELN22) guidelines, determination of *NPM1* mutation status is not only a key step in AML diagnosis and genetic risk stratification, but also essential for MRD monitoring and prognosis assessment. However, the current genotyping methods are unable to meet the requirement of rapid and convenient detection of diverse 4‐bp insertion mutations at *NPM1* gene, leading to false negatives. To address this challenge, we designed and screened crRNAs for the detection of *NPM1* c.863_864 4‐bp insertions. We demonstrated that LwaCas13a/crRNA can tolerate 4‐bp substitution mismatches at positions 9–12 when targeting insertion mutations, while the corresponding indel mismatch in the wild‐type sequence produces only background‐level fluorescence under the optimized assay conditions, enabling comprehensive and highly specific detection of multiple 4‐bp insertions at the same site. By strategically leveraging the specificity defects of the CRISPR/Cas13 system, we enable accurate detection of target sequences and flexible screening of similar variants, thereby improving the assay's adaptability and expanding its clinical utility.

More broadly, we integrated the CRISPR‐Scan detection system with a homemade smart integrated microfluidic chip, rendering it ideal for point‐of‐care applications. It demonstrated a detection sensitivity as low as 10 copies and a detection limit of 0.01% for mutant‐doped samples. In addition, since LwaCas13a targets RNA, the CRISPR‐Scan system has no risk of depleting target DNA. Moreover, the highly sensitive CRISPR‐Scan detection system demonstrates extraordinary MRD monitoring capabilities, with sensitivity comparable to NGS, and effectively predicts the risk of recurrence. Furthermore, we evaluated the performance of the CRISPR‐Scan system in the detection of indels of other cancer‐related genes, such as *KIT*, *BRAF*, and *EGFR*, and confirmed that it can serve as a versatile technique for indel detection, enabling recognition of indel mutations with similar variants. To prevent false positive and false negative results in the detection process, we introduce RPA primers for internal control genes in the CRISPR‐Scan system, enabling duplex amplification and detection. Overall, this approach offers great potential for simple, sensitive, and specific detection of multiplex indels at the same site in settings with limited resources.

## 5 Conclusion

In summary, we developed a detection system named CRISPR‐Scan for detecting multiple indels at the same site based on the mismatch tolerance rules of LwaCas13a. By strategically leveraging the specificity limitations of the LwaCas13a system, we can not only accurately identify indel sequences, but also cover various mutation variants. This enhances the universality of the detection and expands its clinical applicability. The CRISPR‐Scan system demonstrated extremely high sensitivity and could detect *NPM1* c.863_864 4‐bp insertions as low as 0.01%, which is superior to FGS and comparable to NGS. It has an accurate detection effect for MRD and can be used for regular follow‐up and efficacy assessment. By combining the CRISPR‐Scan system with microfluidic chips and a microfluidic device, false positive and false negative results can be avoided, leading to more accurate clinical test results. In conclusion, our CRISPR‐Scan method is not only sensitive, specific, rapid, and convenient, but also cost‐effective. It has significant advantages in bedside detection applications, including cancer classification, efficacy assessment, early warning of drug resistance mutations, and MRD detection, demonstrating great potential in advancing precision cancer medicine.

## Author Contributions

**Linlin Bai**: Conceptualization, Methodology, Software, Data curation, Investigation, Validation, Formal analysis, Visualization, Resources, Writing – original draft, Writing – review & editing. **Bei Wang**: Formal analysis, Funding acquisition, Project administration, Resources, Writing – review & editing. **Mingmin Shi**: Validation, Formal analysis, Writing – review & editing. **Dianwei Liu**: Investigation, Validation. **Huimin Ji**: Investigation, Validation. **Ting Wang**: Funding acquisition, Project administration, Resources. **Ziwei Wang**: Resources, Project administration. **Kaiming Guo**: Validation, Formal analysis. **Xinyang Dou**: Validation. **Chunyan Qian**: Project administration, Resources. **Fang Zhang**: Project administration, Resources, Supervision. **Yongming Wang**: Project administration, Resources, Supervision. **Hongju Mao**: Project administration, Supervision. **Rui Wang**: Conceptualization, Methodology, Resources, Supervision, Funding acquisition, Project administration, Writing – review & editing.

## Declaration of Generative AI Use

During the preparation of this manuscript, the authors used ChatGPT solely to improve the clarity and language of the text. All AI‐assisted output was reviewed and edited by the authors, who take full responsibility for the final content. No AI technologies were used for study design, data analysis, or the generation or modification of figures.

## Conflicts of Interest

The authors declare no conflicts of interest.

## Supporting information




**Supporting File**: advs78229‐sup‐0001‐SuppMat.docx.

## Data Availability

The raw sequencing data generated in this study have been deposited in the Genome Sequence Archive for Human (GSA‐Human, https://ngdc.cncb.ac.cn/gsa‐human/) at the National Genomics Data Center, China National Center for Bioinformation/Beijing Institute of Genomics, Chinese Academy of Sciences. The Sanger sequencing data are available under number HRA020788, whereas the NGS data are available under number HRA020212. All other relevant data are included in the article and its Supporting Information.
