# Supplementary material for: CRISPR‐Scan: Cas13a Mismatch Tolerance Enabled Ultra‐Multiplexed Detection of Same‐Site Indels in Acute Myeloid Leukemia
Source: Adv Sci (Weinh). 2026 Oct 14:e78229. Online ahead of print. doi: 10.1002/advs.78229 (PMC13669218; doi:10.1002/advs.78229)
Supplement: Supplementary file 1 [file ADVS-9999-e78229-s001.docx]

**Supplementary Information**

**CRISPR-Scan: Cas13a Mismatch Tolerance Enabled**

**Ultra-Multiplexed Detection of Same-Site Indels**

**in Acute Myeloid Leukemia**

Linlin Bai^a†^, Bei Wang^b†^, Mingmin Shi^a†^, Dianwei Liu^a^, Huimin Ji^a^, Ting Wang^c^, Ziwei Wang^d^, Kaiming Guo^a^, Xinyang Dou^a^, Chunyan Qian^a,e^, Fang Zhang^f,g^, Yongming Wang^a^, Hongju Mao^a^, Rui Wang^a,h *^

^a^Center for Medical Research and Innovation, Department of Laboratory Medicine, Shanghai Pudong Hospital, Human Phenome Institute, State Key Laboratory of Genetics and Development of Complex Phenotypes, School of Life Sciences, Fudan University, Shanghai 200438, China

^b^State Key Laboratory of Digital Medical Engineering, Department of Otolaryngology Head and Neck Surgery, Zhongda Hospital, School of Medicine, Advanced Institute for Life and Health, Jiangsu Province High-Tech Key Laboratory for Bio-Medical Research, Southeast University, Nanjing, 210096, China

^c^Department of Hematology, Peking University Shenzhen Hospital, Shenzhen Peking University-The Hong Kong University of Science and Technology Medical Center, Shenzhen, 518036, China

^d^Department of Gastroenterology, Shanghai Institute of Pancreatic Diseases, Changhai Hospital; National Key Laboratory of Immunity and Inflammation, Naval Medical University, Shanghai 200433, China

^e^Clinical Laboratory, Linping Campus, The Second Affiliated Hospital of Zhejiang University School of Medicine, Hangzhou 311100, China

^f^College of Biological Science and Engineering, Fuzhou University, Fuzhou, 350108, China

^g^Research Institute for Microbiology and Department of Food Science and Nutrition, The Hong Kong Polytechnic University, Kowloon, Hong Kong, China

^h^International Human Phenome Institutes, Shanghai 200433, China

^*^Corresponding author:

E-mail address: wangr@fudan.edu.cn

^†^These authors contributed equally.

**Table of Contents**

**Materials and Methods**..................................................................................................................4

The crRNA and target RNA preparation..........................................................................................4

Preparation of mutant mock DNA samples......................................................................................4

First-generation sequencing (FGS) and next-generation sequencing (NGS)....................................4

**Figure S1** Schematic illustration of the pathological features of AML and the principle of the CRISPR-Scan detection strategy......................................................................................................6

**Figure S2** Position-dependent effects of 4-nt indels on LwaCas13a trans-cleavage activity for the PA target...........................................................................................................................................7

**Figure S3** Position and number dependent tolerance of LwaCas13a to consecutive substitution mismatches in the PA target.............................................................................................................8

**Figure S4** Position-dependent effects of 4-nt indels on LwaCas13a trans-cleavage activity for the BRAF target.....................................................................................................................................9

**Figure S5** Position and number dependent tolerance of LwaCas13a to consecutive substitution mismatches in the BRAF target......................................................................................................10

**Figure S6** Number of mismatched bases between CCTG-crRNA and TCTG-crRNA in relation to the top 11 highest frequency types of *NPM1* c.863_864 4-bp insertions.........................................11

**Figure S7** Endpoint fluorescence intensity of CRISPR-Scan system for the top 11 highest frequency types of *NPM1* c.863_864 4-bp insertions with LwaCas13a/crRNA 9-12R..................12

**Figure S8** Molecular dynamics data of LwaCas13a/crRNA 9-12R with *NPM1*_WT, *NPM1*_insCCTG, and *NPM1*_insCAGA RNA templates..............................................................13

**Figure S9** Molecular dynamics data of LwaCas13a/crRNA 9-12R with *NPM1*_WT, *NPM1*_insCCTG, and *NPM1*_insCAGA RNA templates..............................................................14

**Figure S10** Sensitivity of the CRISPR‑Scan system for NPM1 c.863_864insTCTG and NPM1 c.863_864insCATG mutations with plasmid templates.................................................................15

**Figure S11** Sensitivity of the CRISPR‑Scan system for NPM1 c.863_864insTAAG and NPM1 c.863_864insCAGA mutations with plasmid templates.................................................................16

**Figure S12** Detection of AML clinical samples genomic DNA using the CRISPR-Scan system...17

**Figure S13** Sensitivity evaluation of CRISPR-Scan system with genomic DNA templates..........18

**Figure S14** Visualization of CRISPR-Scan detection results for 120 AML clinical samples.........20

**Figure S15** Detection results of the NPM1 c.863_864 4-bp insertions in AML clinical samples by FGS, NGS, and CRISPR-Scan system...........................................................................................24

**Figure S16** FGS and NGS analysis results of eight bone marrow samples from Patient-MRD-1 from disease onset to 14 months after onset....................................................................................25

**Figure S17** FGS, NGS, and CRISPR-Scan detection results of Patient-MRD-2............................26

**Figure S18** FGS, NGS, and CRISPR-Scan detection results of Patient-MRD-3............................27

**Figure S19** FGS, NGS, and CRISPR-Scan detection results of Patient-MRD-4............................28

**Figure S20** Diagram of the home-made microfluidic device.........................................................29

**Figure S21** Diagram of the integrated microfluidic chip................................................................30

**Figure S22** Visualization results of CRISPR-Scan detection using the microfluidic device for 40 of the 120 AML clinical samples....................................................................................................31

**Table S1** Oligonucleotide sequences for the preparation of *NPM1* RNA templates with mismatches and indels....................................................................................................................32

**Table S2** Oligonucleotide sequences for the preparation of PA RNA templates with mismatches and indels.......................................................................................................................................34

**Table S3** Oligonucleotide sequences for the preparation of *BRAF* RNA templates with mismatches and indels.......................................................................................................................................36

**Table S4** Sequence information for in vitro transcription of *NPM1,* PA and *BRAF* LwaCas13a crRNA............................................................................................................................................38

**Table S5** Oligonucleotide sequences for the preparation of *NPM1*-WT and *NPM1*-insCCTG template RNAs...............................................................................................................................39

**Table S6** Oligonucleotide sequences for the preparation of RNA templates for 40 types of *NPM1* c.863_864 4-bp insertions..............................................................................................................40

**Table S7** Oligonucleotide sequences for preparation of RNA templates for the top 11 highest frequency types of *NPM1* c.863_864 4-bp insertions.....................................................................42

**Table S8** RPA Primer sequences used in CRISPR-Scan system....................................................43

**Table S9** Primer sequences for FGS...............................................................................................44

**Table S10** Primer sequences for Next-generation sequencing.......................................................45

**Table S11** NPM1 mutation VAF and bone marrow blast percentage during MRD monitoring.....47

**Table S12** Oligonucleotide sequences used for the preparation of *KIT, BRAF, EGFR, GAPDH* LwaCas13a crRNA and corresponding template RNAs.................................................................48

Sequence information of plasmids.................................................................................................49

**Materials and Methods**

**The crRNA and target RNA preparation**

Clustered regularly interspaced short palindromic repeats RNA (crRNA) was synthesized and purified with T7 Transcription KIT. Firstly, the template was amplified via PCR assay. Then, 2 μL of T7 polymerase, 10 μL of NTP buffer and 4 μL of the PCR product were mixed, and DEPC water was added to make up a 30 μL transcription system. This mixture was then incubated at 37 °C for 24 h to transcribe the DNA template into RNA. Next, 50 μL reaction system including DNase I 5 μL ,10 × DNase I buffer 5 μL, RNase inhibitor 20 U was heated at 37 °C for 30 min to ensure complete digestion of the DNA template. Then, the obtained RNA products were purified using HiPure RNA Pure Micro KIT, following the operation instructions. Finally, the RNA was eluted into RNase-free water and preserved at −80 °C refrigerator for further use.

**Preparation of mutant mock DNA samples**

For plasmid mutant mock DNA Samples preparation, Mutant and wild-type (WT) plasmid were mixed at various ratios, with the *NPM1* c.863_864insCCTG, *NPM1* c.863_864insTCTG, *NPM1* c.863_864insCATG, *NPM1* c.863_864insTAAG or *NPM1* c.863_864insCAGA mutation comprising 100%~0.001% (i.e., 10 copies of the mutation template in a total of 1e6 templates) of the total template pool. A total of 1e6 copies of the mixed plasmid DNA was used as template for CRISPR-Scan detection.

For genomic DNA Mutation-Mocked Samples preparation, genomic DNA containing 32.9% *NPM1* c.863_864insTCTG mutations from acute myeloid leukemia (AML) patient samples was mixed with wild-type genomic DNA from healthy individuals to prepare genomic DNA mutation-doped samples with mutation concentrations ranging from 32.9% to 0.001%. A total of 80 ng of genomic DNA was used as template for CRISPR-Scan detection.

**First-generation sequencing (FGS) and next-generation sequencing (NGS)**

For FGS, the extracted DNA was amplified by PCR. The amplification system contained Takara Taq HS (5 U/μL) 0.25 μL, 10 × PCR Buffer (Mg^2+^ plus) 5 μL, dNTP Mixture (each 2.5 mM) 5 μL, Template 3 μL, primer F/R 400 nM, and DEPC water up to 50 μL. The PCR procedure was 95 °C 5 min, (95 °C 20s, 55 °C 30s, 72 °C 30s) × 40 cycles, and 72 °C 5 min. 20 µl PCR products were sent for FGS by Sangon Biotech (Shanghai, China).

For NGS, the extracted DNA was firstly amplified by PCR with primers containing barcodes. The amplification system contained 2 × PCR buffer for KOD FX 12.5 μL, 2 mM dNTPs 5 μL, 10 μM Primer F/R 0.75 μL, Template 5 μL, KOD FX (1.0 U/μL) 0.5 μL, and DEPC water up to 25 μL. First round PCR procedure was 94 °C 2 min, (98 °C 10 s, 55 °C 30 s, 68 °C 30 s) × 25 cycles, and 68 °C 7 min. In the second round of PCR, different barcode primers were used to amplify the amplicons of samples. The amplification system and procedure were as follows: 2 × PCR buffer for KOD FX 25 μL, 2 mM dNTPs 10 μL, 10 μM Primer F/R 1.5 μL (Specific primer sequences were shown in Table S11), 10 μL Amplicons of the first round PCR, KOD FX (1.0 U/μL) 1 μL, and DEPC water up to 50 μL. The second round PCR procedure was 94 °C 2 min, (98 °C 10 s, 57 °C 30 s, 68 °C 30 s) × 15 cycles, 68 °C 7 min. The PCR products were purified by TIANgel Midi Purification Kit and sent to Sequanta Technologies (Shanghai, China) for NGS.

Figure S1. Schematic illustration of the pathological features of AML and the principle of the CRISPR-Scan detection strategy. In AML patients, malignant transformation of myeloid cells leads to excessive proliferation of immature cells in the bone marrow, which crowd out healthy blood cells and inhibit normal hematopoiesis. *NPM1*-mutant AML represents the most common AML subtype, accounting for 30-35% of adult AML cases, and is characterized by a 4-bp insertion at c.863_864 in exon 12. More than 100 types of c.863_864 4-bp insertions in the *NPM1* gene have been reported, of which 11 account for over 97% of *NPM1*-mutant AML cases, with c.863_864insTCTG (76.4%), insCATG (9.2%), and insCCTG (7.2%) representing the most prevalent. The CRISPR-Scan detection method was developed by leveraging the tolerance of LwaCas13a to up to four continuous mismatches and its intolerance to indels, thereby enabling detection of ultra-multiplex *NPM1* 4-bp insertion mutations with a single crRNA. Created in BioRender. Bai, L. (2026) https://BioRender.com/iyzehlh.

**Figure S2.** Position-dependent effects of 4-nt indels on LwaCas13a trans-cleavage activity for the PA target. Normalized endpoint fluorescence signals at 30 min of the LwaCas13a *trans*-cleavage assay using PA target RNAs containing a 4-nt deletion or insertion at different spacer positions. The fluorescence signals were normalized to the signal obtained with the perfectly matched target (Match). Data are shown as means ± s.d. (n = 3 technical replicates). NTC, no-template control.

**Figure S3.** Position and number dependent tolerance of LwaCas13a to consecutive substitution mismatches in the PA target. Normalized endpoint fluorescence signals at 30 min of the LwaCas13a *trans*-cleavage assay using PA target RNAs containing 2-, 3-, or 4-nt consecutive substitution mismatches across seven spacer regions (LR1–LR7). The fluorescence signals were normalized to the signal obtained with the perfectly matched target (PM). Data grouped by mismatch location (Ln) and mismatch number (Qm) are illustrated as box plots. Center lines indicate medians, boxes indicate interquartile ranges, whiskers indicate lower and upper adjacent values, black points indicate individual observations, and open squares indicate means. The upper-right inset shows the results of two-way ANOVA. Bar-plot data are shown as means ± s.d. (n = 3 technical replicates). SOV, source of variation; NC, negative control; PM, perfectly matched target; *****p* < 0.0001.

**Figure S4.** Position-dependent effects of 4-nt indels on LwaCas13a trans-cleavage activity for the BRAF target. Normalized endpoint fluorescence signals at 30 min of the LwaCas13a *trans*-cleavage assay using *BRAF* target RNAs containing a 4-nt deletion or insertion at different spacer positions. The fluorescence signals were normalized to the signal obtained with the perfectly matched target (Match). Data are shown as means ± s.d. (n = 3 technical replicates). NTC, no-template control.

**Figure S5.** Position and number dependent tolerance of LwaCas13a to consecutive substitution mismatches in the *BRAF* target. Normalized endpoint fluorescence signals at 30 min of the LwaCas13a *trans*-cleavage assay using *BRAF* target RNAs containing 2-, 3-, or 4-nt consecutive substitution mismatches across seven spacer regions (LR1–LR7). The fluorescence signals were normalized to the signal obtained with the perfectly matched target (PM). Data grouped by mismatch location (Ln) and mismatch number (Qm) are illustrated as box plots. Center lines indicate medians, boxes indicate interquartile ranges, whiskers indicate lower and upper adjacent values, black points indicate individual observations, and open squares indicate means. The upper-right inset shows the results of two-way ANOVA. Bar-plot data are shown as means ± s.d. (n = 3 technical replicates). SOV, source of variation; NC, negative control; PM, perfectly matched target; *****p* < 0.0001.

Figure S6. Number of mismatched bases between CCTG-crRNA and TCTG-crRNA in relation to the top 11 highest frequency types of *NPM1* c.863_864 4-bp insertions. a, Table summarizing the number of mismatched bases. b, Bar chart showing the number of mismatched bases. The CCTG-crRNA exhibits fewer mismatches with the top 11 common NPM1 c.863_864 4-bp insertions, indicating higher recognition activity.

Figure S7. Endpoint fluorescence intensity of CRISPR-Scan system for the top 11 highest frequency types of *NPM1* c.863_864 4-bp insertions with LwaCas13a/crRNA 9-12R. From left to right: TCTG, CATG, CCTG, CCAG, CCGG, CTTG, TATG, TCGG, TAAG, CAGG, and CAGA, with WT as the control. WT, wild-type. Data shown are means ± s.d. (*n* = 3 technical replicates). The indicated groups were compared with the WT. Statistical significance was assessed using unpaired two-tailed Student’s *t*-tests. A *p* value < 0.05 was considered statistically significant; ns, not significant.

Figure S8. Molecular dynamics analysis of LwaCas13a/crRNA 9-12R with *NPM1*_WT, *NPM1*_insCCTG, and *NPM1*_insCAGA RNA templates. a-c, Root Mean Square Deviation (RMSD) curves of LwaCas13a/crRNA 9-12R with *NPM1*_WT, *NPM1*_insCCTG, and *NPM1*_insCAGA RNA templates over time. d-f, Change in the number of hydrogen bonds between LwaCas13a/crRNA 9-12R and *NPM1*_WT, *NPM1*_insCCTG, and *NPM1*_insCAGA RNA templates over time.

**Figure S9.** Molecular dynamics analysis of LwaCas13a/crRNA 9-12R with *NPM1*_WT, *NPM1*_insCCTG, and *NPM1*_insCAGA RNA templates. **a-c,** Interaction diagrams between LwaCas13a/crRNA 9-12R and *NPM1*_WT, *NPM1*_insCCTG, and *NPM1*_insCAGA RNA templates. **d-f,** Average binding free energy between LwaCas13a/crRNA 9-12R and *NPM1*_WT, *NPM1*_insCCTG, and *NPM1*_insCAGA RNA templates.

**Figure S10.** Sensitivity evaluation of the CRISPR-Scan system with plasmid templates. a, Representative real-time fluorescence curves of the integrated one-pot CRISPR-Scan system for the detection of the *NPM1* c.863_864insTCTG mutation using 1e6 copies of plasmid as a template, incubated at 39 °C for 30 min. b, Endpoint fluorescence values and visualization results for c.863_864insTCTG mutation detection in (a). c, Representative real-time fluorescence curves of the integrated one-pot CRISPR-Scan system for detection of the *NPM1* c.863_864insCATG mutation, using 1e6 copies of plasmid as a template, incubated at 39 °C for 30 min. d, Endpoint fluorescence values and visualization results for c.863_864insCATG mutation detection in (c). WT, wild-type; NTC, no-template control. Data shown are means ± s.d. (*n* = 3 technical replicates). The indicated groups were compared with the NTC. Statistical significance was assessed using unpaired two-tailed Student’s *t*-tests. A *p* value < 0.05 was considered statistically significant; ns, not significant.

**Figure S11. Analytical sensitivity of the CRISPR-Scan system using plasmid templates.** Endpoint fluorescence intensities and corresponding visual readouts of the integrated one-pot CRISPR-Scan system for detecting the *NPM1* c.863_864insTAAG (a) and c.863_864insCAGA (b) mutations in plasmid mixtures with variant allele frequencies (VAFs) ranging from 1% to 0.001% after incubation at 39 °C for 30 min. WT, wild-type; NTC, no-template control. Data are shown as means ± s.d. (n = 3 technical replicates). The indicated groups were compared with NTC. Statistical significance was assessed using unpaired two-tailed Student’s *t*-tests. A *p* value < 0.05 was considered statistically significant; ns, not significant.

Figure S12. Detection of AML clinical samples genomic DNA using the CRISPR-Scan system. Endpoint fluorescence values for the detection of NPM1 c.863_864 4-bp insertion mutations in three AML patient samples (a–c), using varying amounts of blood genomic DNA as templates. Data are presented as means ± s.d.(n = 3 technical replicates).

Figure S13. Sensitivity evaluation of the CRISPR-Scan system using genomic DNA templates. a, Representative real-time fluorescence curves of the integrated one-pot CRISPR-Scan system for detecting the *NPM1* c.863_864insTCTG mutation in genomic DNA samples with defined variant allele frequencies (VAFs). The samples were prepared by mixing patient-derived mutant genomic DNA with healthy wild-type genomic DNA. Reactions were incubated at 39 °C for 30 min. b, Endpoint fluorescence intensities and corresponding visual readouts for c.863_864insTCTG detection in (a). c, Weighted linear regression analysis of endpoint fluorescence intensity against log₁₀[VAF (%)] for c.863_864insTCTG. d, Representative real-time fluorescence curves of the integrated one-pot CRISPR-Scan system for detecting the *NPM1* c.863_864insCCTG mutation in genomic DNA samples with defined VAFs, prepared and analyzed as described in (a). e, Endpoint fluorescence intensities and corresponding visual readouts for c.863_864insCCTG detection in (d). f, Weighted linear regression analysis of endpoint fluorescence intensity against log₁₀[VAF (%)] for c.863_864insCCTG. For panels c and f, weights were set to the inverse square of the SD (1/SD²) at each VAF. WT, wild-type; NTC, no-template control. Data are shown as means ± s.d. (n = 3 technical replicates). For panels b and e, the indicated groups were compared with NTC. Statistical significance was assessed using unpaired two-tailed Student’s *t*-tests. A *p* value < 0.05 was considered statistically significant; ns, not significant.

**Figure S14.** Visualization of CRISPR-Scan detection results for 120 AML clinical samples. All reaction samples were photographed under blue light.

Figure S15. Detection results of *NPM1* c.863_864 4-bp insertions in AML clinical samples by FGS, NGS, and CRISPR-Scan system. FGS results were shown in electrophoresis peak map, with mutated bases underlined in red. Mutation types and frequencies identified by NGS are shown in the upper right. Visualization of CRISPR-Scan detection results is shown on the right.

Figure S16. FGS and NGS analysis results of eight bone marrow samples from Patient-MRD-1 from disease onset to 14 months after onset. FGS results were shown in electrophoresis peak maps, with mutated bases underlined in red. Mutation types and frequencies identified by NGS were shown in the upper right.

Figure S17. FGS, NGS, and CRISPR-Scan detection results of Patient-MRD-2. a, Representative real-time fluorescence curves of the CRISPR-Scan system for detection of *NPM1* c.863_864 4-bp insertions in samples from Patient-MRD-2 and a healthy control (HC). b, FGS results were shown in electrophoresis peak maps, with mutated bases underlined in red. Mutation types and frequencies identified by NGS were shown in the upper right.

Figure S18. FGS, NGS, and CRISPR-Scan detection results of Patient-MRD-3. a, Representative real-time fluorescence curves of the CRISPR-Scan system for detection of *NPM1* c.863_864 4-bp insertions in samples from Patient-MRD-3 and a healthy control (HC). b, FGS results were shown in electrophoresis peak maps, with mutated bases underlined in red. Mutation types and frequencies identified by NGS were shown in the upper right.

Figure S19. FGS, NGS, and CRISPR-Scan detection results of Patient-MRD-4. a, Representative real-time fluorescence curves of the CRISPR-Scan system for detection of *NPM1* c.863_864 4-bp insertions in samples from Patient-MRD-4 and a healthy control (HC). b, FGS results were shown in electrophoresis peak maps, with mutated bases underlined in red. Mutation types and frequencies identified by NGS were shown in the upper right.

**Figure S20.** Photograph of the home-made microfluidic device. The photograph was taken with a smartphone. The device features a rectangular appearance incorporating a temperature sensor and a heating block at the base. The temperature sensor provides real-time feedback on the heating block temperature, which is displayed on the dashboard. The microfluidic chip is placed on the heating block and illuminated by surrounding blue LED lights. The front of the enclosure includes a switch and a display dashboard. The top of the device houses a phone holder equipped with a filter. During operation, the device is powered by a mobile power unit. Pressing the switch activates the blue LEDs. The target temperature is then set via the dashboard. After placing the microfluidic chip on the heating block, the mobile signal reading system is initiated for real-time monitoring and curve output.

Figure S21. Photograph of the integrated microfluidic chip. The photograph was taken with a smartphone.

Figure S22. Visualization results of CRISPR-Scan detection using the microfluidic device for 40 of the 120 AML clinical samples. In each device, the upper-left, upper-right, and lower chambers correspond to the positive control, negative control, and clinical sample, respectively. For each sample, 80 ng of genomic DNA was used.

**Table S1 Oligonucleotide sequences for the preparation of *NPM1* RNA templates**

with mismatches and indels

| **Name** | ***Sequence (5′-3′)** |
| --- | --- |
| *NPM1*-Fm1 | TTTGTAATTAGAGGTGATGAAGTCAGACAAATCGCTCCACTCCTACATCCTATTCAAGA |
| *NPM1*-T7-Rm1 | GAAATTAATACGACTCACTATAGGGTCATTTAATTTAGTACACTCCATAACAAGACTTC |
| *NPM1*-CCTG | CTCCTACATCCTATTCAAGATCTCTGCCTGGCAGTGGAGGAAGTCTTGTTATGGAGTGT |
| LR1 Tat Q2 | CTCCTACATCCTATTCAACTTCTCTGCCTGGCAGTGGAGGAAGTCTTGTTATGGAGTGT |
| LR1 Tta Q3: | CTCCTACATCCTATTCAACTACTCTGCCTGGCAGTGGAGGAAGTCTTGTTATGGAGTGT |
| LR1 Tcg Q4 | CTCCTACATCCTATTCAACTAGTCTGCCTGGCAGTGGAGGAAGTCTTGTTATGGAGTGT |
| LR2 Tcg Q2 | CTCCTACATCCTATTCAAGATCAGTGCCTGGCAGTGGAGGAAGTCTTGTTATGGAGTGT |
| LR2 Tta Q3 | CTCCTACATCCTATTCAAGATCAGAGCCTGGCAGTGGAGGAAGTCTTGTTATGGAGTGT |
| LR2 Tgc Q4 | CTCCTACATCCTATTCAAGATCAGACCCTGGCAGTGGAGGAAGTCTTGTTATGGAGTGT |
| LR3 Tcg Q2 | CTCCTACATCCTATTCAAGATCTCTGGGTGGCAGTGGAGGAAGTCTTGTTATGGAGTGT |
| LR3 Tta Q3 | CTCCTACATCCTATTCAAGATCTCTGGGAGGCAGTGGAGGAAGTCTTGTTATGGAGTGT |
| LR3 Tgc Q4 | CTCCTACATCCTATTCAAGATCTCTGGGACGCAGTGGAGGAAGTCTTGTTATGGAGTGT |
| LR4 Tcg Q2 | CTCCTACATCCTATTCAAGATCTCTGCCTGCGAGTGGAGGAAGTCTTGTTATGGAGTGT |
| LR4 Tat Q3 | CTCCTACATCCTATTCAAGATCTCTGCCTGCGTGTGGAGGAAGTCTTGTTATGGAGTGT |
| LR4 Tgc Q4 | CTCCTACATCCTATTCAAGATCTCTGCCTGCGTCTGGAGGAAGTCTTGTTATGGAGTGT |
| LR5 Tgc Q2 | CTCCTACATCCTATTCAAGATCTCTGCCTGGCAGACGAGGAAGTCTTGTTATGGAGTGT |
| LR5 Tgc Q3 | CTCCTACATCCTATTCAAGATCTCTGCCTGGCAGACCAGGAAGTCTTGTTATGGAGTGT |
| LR5 Tat Q4 | CTCCTACATCCTATTCAAGATCTCTGCCTGGCAGACCTGGAAGTCTTGTTATGGAGTGT |
| LR6 Tgc Q2 | CTCCTACATCCTATTCAAGATCTCTGCCTGGCAGTGGACCAAGTCTTGTTATGGAGTGT |
| LR6 Tat Q3 | CTCCTACATCCTATTCAAGATCTCTGCCTGGCAGTGGACCTAGTCTTGTTATGGAGTGT |
| LR6 Tat Q4 | CTCCTACATCCTATTCAAGATCTCTGCCTGGCAGTGGACCTTGTCTTGTTATGGAGTGT |
| LR7 Tta Q2 | CTCCTACATCCTATTCAAGATCTCTGCCTGGCAGTGGAGGAACACTTGTTATGGAGTGT |
| LR7 Tcg Q3 | CTCCTACATCCTATTCAAGATCTCTGCCTGGCAGTGGAGGAACAGTTGTTATGGAGTGT |
| LR7 Tta Q4 | CTCCTACATCCTATTCAAGATCTCTGCCTGGCAGTGGAGGAACAGATGTTATGGAGTGT |
| *NPM1*-del1-4 | CTCCTACATCCTATTCAATCTGCCTGGCAGTGGAGGAAGTCTTGTTATGGAGTGT |
| *NPM1*-del5-8 | CTCCTACATCCTATTCAAGATCCCTGGCAGTGGAGGAAGTCTTGTTATGGAGTGT |
| *NPM1*-del9-12 | CTCCTACATCCTATTCAAGATCTCTGGCAGTGGAGGAAGTCTTGTTATGGAGTGT |
| *NPM1*-del13-16 | CTCCTACATCCTATTCAAGATCTCTGCCTGTGGAGGAAGTCTTGTTATGGAGTGT |
| *NPM1*-del17-20 | CTCCTACATCCTATTCAAGATCTCTGCCTGGCAGGGAAGTCTTGTTATGGAGTGT |
| *NPM1*-del21-24 | CTCCTACATCCTATTCAAGATCTCTGCCTGGCAGTGGAGTCTTGTTATGGAGTGT |
| *NPM1*-del25-28 | CTCCTACATCCTATTCAAGATCTCTGCCTGGCAGTGGAGGAATGTTATGGAGTGT |
| *NPM1*-in5-8 | CCTACATCCTATTCAAGATCTAGTTCTGCCTGGCAGTGGAGGAAGTCTTGTTATGGAGT |
| *NPM1*-in9-12 | CCTACATCCTATTCAAGATCTCTGTAGTCCTGGCAGTGGAGGAAGTCTTGTTATGGAGT |
| *NPM1*-in13-16 | CCTACATCCTATTCAAGATCTCTGCCTGTAGTGCAGTGGAGGAAGTCTTGTTATGGAGT |
| *NPM1*-in17-20 | CCTACATCCTATTCAAGATCTCTGCCTGGCAGTAGTTGGAGGAAGTCTTGTTATGGAGT |
| *NPM1*-in21-24 | CCTACATCCTATTCAAGATCTCTGCCTGGCAGTGGATAGTGGAAGTCTTGTTATGGAGT |
| *NPM1*-in25-28 | CCTACATCCTATTCAAGATCTCTGCCTGGCAGTGGAGGAATAGTGTCTTGTTATGGAGT |

* The T7 promoter sequence is labeled in green. Mismatch sites are highlighted in red font. *NPM1*-Fm and *NPM1*-T7-Rm are the forward and reverse primers, and the oligonucleotide sequences containing mutations or indels serve as the amplification templates. The forward primer, reverse primer, and amplification template undergo a PCR amplification reaction. The resulting products are then transcribed using T7 RNA polymerase, followed by RNA purification and quantification to obtain RNA templates containing mutations or indels.

**Table S2 Oligonucleotide sequences for the preparation of PA RNA templates**

with mismatches and indels

| **Name** | ***Sequence (5′-3′)** |
| --- | --- |
| PA-Fm | GAAATTAATACGACTCACTATAGGGTACAATCGCTTCTTTAGGTCACTTCTATTCAAGA |
| PA-Match | tcctacATCCTGTGCGTTACCAGACGATGTTCGCCTCGATCTTGAATAGAAGTGACCTA |
| PALR1 Tta Q2 | tcctacATCCTcaGCGTTACCAGACGATGTTCGCCTCGATCTTGAATAGAAGTGACCTA |
| PALR1 Tgc Q3 | tcctacATCCTcacCGTTACCAGACGATGTTCGCCTCGATCTTGAATAGAAGTGACCTA |
| PALR1 Tcg Q4 | tcctacATCCTcacgGTTACCAGACGATGTTCGCCTCGATCTTGAATAGAAGTGACCTA |
| PALR2 Tta Q2 | tcctacATCCTGTGCcaTACCAGACGATGTTCGCCTCGATCTTGAATAGAAGTGACCTA |
| PALR2 Tta Q3 | tcctacATCCTGTGCcaaACCAGACGATGTTCGCCTCGATCTTGAATAGAAGTGACCTA |
| PALR2 Tat Q4 | tcctacATCCTGTGCcaatCCAGACGATGTTCGCCTCGATCTTGAATAGAAGTGACCTA |
| PALR3 Tcg Q2 | tcctacATCCTGTGCGTTAggAGACGATGTTCGCCTCGATCTTGAATAGAAGTGACCT |
| PALR3 Tat Q3 | tcctacATCCTGTGCGTTAggtGACGATGTTCGCCTCGATCTTGAATAGAAGTGACCT |
| PALR3 Tgc Q4 | tcctacATCCTGTGCGTTAggtcACGATGTTCGCCTCGATCTTGAATAGAAGTGACCT |
| PALR4 Tcg Q2 | tcctacATCCTGTGCGTTACCAGtgGATGTTCGCCTCGATCTTGAATAGAAGTGACCTA |
| PALR4 Tgc Q3 | tcctacATCCTGTGCGTTACCAGtgcATGTTCGCCTCGATCTTGAATAGAAGTGACCTA |
| PALR4 Tat Q4 | tcctacATCCTGTGCGTTACCAGtgctTGTTCGCCTCGATCTTGAATAGAAGTGACCTA |
| PALR5 Tgc Q2 | tcctacATCCTGTGCGTTACCAGACGAacTTCGCCTCGATCTTGAATAGAAGTGACCTA |
| PALR5 Tta Q3 | tcctacATCCTGTGCGTTACCAGACGAacaTCGCCTCGATCTTGAATAGAAGTGACCTA |
| PALR5 Tta Q4 | tcctacATCCTGTGCGTTACCAGACGAacaaCGCCTCGATCTTGAATAGAAGTGACCTA |
| PALR6 Tgc Q2 | tcctacATCCTGTGCGTTACCAGACGATGTTgcCCTCGATCTTGAATAGAAGTGACCTA |
| PALR6 Tcg Q3 | tcctacATCCTGTGCGTTACCAGACGATGTTgcgCTCGATCTTGAATAGAAGTGACCTA |
| PALR6 Tcg Q4 | tcctacATCCTGTGCGTTACCAGACGATGTTgcggTCGATCTTGAATAGAAGTGACCTA |
| PALR7 Tcg Q2 | tcctacATCCTGTGCGTTACCAGACGATGTTCGCCagGATCTTGAATAGAAGTGACCTA |
| PALR7 Tgc Q3 | tcctacATCCTGTGCGTTACCAGACGATGTTCGCCagcATCTTGAATAGAAGTGACCTA |
| PALR7 Tat Q4 | tcctacATCCTGTGCGTTACCAGACGATGTTCGCCagctTCTTGAATAGAAGTGACCTA |
| PA-del1-4 | tcctacATCCTGTTACCAGACGATGTTCGCCTCGATCTTGAATAGAAGTGACCTA |
| PA-del5-8 | tcctacATCCTGTGCCCAGACGATGTTCGCCTCGATCTTGAATAGAAGTGACCTA |
| PA-del9-12 | tcctacATCCTGTGCGTTAACGATGTTCGCCTCGATCTTGAATAGAAGTGACCTA |
| PA-del13-16 | tcctacATCCTGTGCGTTACCAGTGTTCGCCTCGATCTTGAATAGAAGTGACCTA |
| PA-del17-20 | tcctacATCCTGTGCGTTACCAGACGACGCCTCGATCTTGAATAGAAGTGACCTA |
| PA-del21-24 | tcctacATCCTGTGCGTTACCAGACGATGTTTCGATCTTGAATAGAAGTGACCTA |
| PA-del25-28 | tcctacATCCTGTGCGTTACCAGACGATGTTCGCCTCTTGAATAGAAGTGACCTA |
| PA-in5-8 | tcctacAGTGCTCCTGTTACCAGACGATGTTCGCCTCGATCTTGAATAGAAGTGACCTA |
|  |  |
| PA-in9-12 | tcctacAGTGCGTTATCCTCCAGACGATGTTCGCCTCGATCTTGAATAGAAGTGACCTA |
| PA-in13-16 | tcctacAGTGCGTTACCAGTCCTACGATGTTCGCCTCGATCTTGAATAGAAGTGACCTA |
| PA-in17-20 | tcctacAGTGCGTTACCAGACGATCCTTGTTCGCCTCGATCTTGAATAGAAGTGACCTA |
| PA-in21-24 | tcctacAGTGCGTTACCAGACGATGTTTCCTCGCCTCGATCTTGAATAGAAGTGACCTA |
| PA-in25-28 | tcctacAGTGCGTTACCAGACGATGTTCGCCTCCTTCGATCTTGAATAGAAGTGACCTA |

* The T7 promoter sequence is labeled in green. Mismatch sites are highlighted in red. The forward and reverse primers serve as templates for each other during PCR amplification. The resulting products are then transcribed using T7 RNA polymerase, followed by RNA purification and quantification to obtain RNA templates containing mutations or indels.

**Table S3 Oligonucleotide sequences for the preparation of *BRAF* RNA templates**

with mismatches and indels

| Name | *Sequence (5′-3′) |
| --- | --- |
| *BRAF*-Fm | GAAATTAATACGACTCACTATAGGGTACAATCGCTTCTTTAGGTCACTTCTATTCAAGA |
| *BRAF*-Match | tcctacATCCTATGAAAATGTTGAATGCTCAGCAGTTACTCTTGAATAGAAGTGACCTA |
| *BRAF*LR1 Tta Q2 | tcctacATCCTTAGAAAATGTTGAATGCTCAGCAGTTACTCTTGAATAGAAGTGACCTA |
| *BRAF*LR1 Tgc Q3 | tcctacATCCTTACAAAATGTTGAATGCTCAGCAGTTACTCTTGAATAGAAGTGACCTA |
| *BRAF*LR1 Tat Q4 | tcctacATCCTTACTAAATGTTGAATGCTCAGCAGTTACTCTTGAATAGAAGTGACCTA |
| *BRAF*LR2 Tat Q2 | tcctacATCCTATGATTATGTTGAATGCTCAGCAGTTACTCTTGAATAGAAGTGACCTA |
| *BRAF*LR2 Tat Q3 | tcctacATCCTATGATTTTGTTGAATGCTCAGCAGTTACTCTTGAATAGAAGTGACCTA |
| *BRAF*LR2 Tta Q4 | tcctacATCCTATGATTTAGTTGAATGCTCAGCAGTTACTCTTGAATAGAAGTGACCTA |
| *BRAF*LR3 Tta Q2 | tcctacATCCTATGAAAATCATGAATGCTCAGCAGTTACTCTTGAATAGAAGTGACCTA |
| *BRAF*LR3 Tta Q3 | tcctacATCCTATGAAAATCAAGAATGCTCAGCAGTTACTCTTGAATAGAAGTGACCTA |
| *BRAF*LR3 Tgc Q4 | tcctacATCCTATGAAAATCAACAATGCTCAGCAGTTACTCTTGAATAGAAGTGACCTA |
| *BRAF*LR4 Tat Q2 | tcctacATCCTATGAAAATGTTGTTTGCTCAGCAGTTACTCTTGAATAGAAGTGACCTA |
| *BRAF*LR4 Tta Q3 | tcctacATCCTATGAAAATGTTGTTAGCTCAGCAGTTACTCTTGAATAGAAGTGACCTA |
| *BRAF*LR4 Tgc Q4 | tcctacATCCTATGAAAATGTTGTTACCTCAGCAGTTACTCTTGAATAGAAGTGACCTA |
| *BRAF*LR5 Tta Q2 | tcctacATCCTATGAAAATGTTGAATGGACAGCAGTTACTCTTGAATAGAAGTGACCTA |
| *BRAF*LR5 Tcg Q3 | tcctacATCCTATGAAAATGTTGAATGGAGAGCAGTTACTCTTGAATAGAAGTGACCTA |
| *BRAF*LR5 Tat Q4 | tcctacATCCTATGAAAATGTTGAATGGAGTGCAGTTACTCTTGAATAGAAGTGACCTA |
| *BRAF*LR6 Tcg Q2 | tcctacATCCTATGAAAATGTTGAATGCTCACGAGTTACTCTTGAATAGAAGTGACCTA |
| *BRAF*LR6 Tat Q3 | tcctacATCCTATGAAAATGTTGAATGCTCACGTGTTACTCTTGAATAGAAGTGACCTA |
| *BRAF*LR6 Tgc Q4 | tcctacATCCTATGAAAATGTTGAATGCTCACGTCTTACTCTTGAATAGAAGTGACCTA |
| *BRAF*LR7 Tta Q2 | tcctacATCCTATGAAAATGTTGAATGCTCAGCAGAAACTCTTGAATAGAAGTGACCTA |
| *BRAF*LR7 Tat Q3 | tcctacATCCTATGAAAATGTTGAATGCTCAGCAGAATCTCTTGAATAGAAGTGACCTA |
| *BRAF*LR7 Tcg Q4 | tcctacATCCTATGAAAATGTTGAATGCTCAGCAGAATGTCTTGAATAGAAGTGACCTA |
| *BRAF*-del1-4 | tcctacATCCTAAATGTTGAATGCTCAGCAGTTACTCTTGAATAGAAGTGACCTA |
| *BRAF*-del5-8 | tcctacATCCTATGAGTTGAATGCTCAGCAGTTACTCTTGAATAGAAGTGACCTA |
| *BRAF*-del9-12 | tcctacATCCTATGAAAATAATGCTCAGCAGTTACTCTTGAATAGAAGTGACCTA |
| *BRAF*-del13-16 | tcctacATCCTATGAAAATGTTGCTCAGCAGTTACTCTTGAATAGAAGTGACCTA |
| *BRAF*-del17-20 | tcctacATCCTATGAAAATGTTGAATGGCAGTTACTCTTGAATAGAAGTGACCTA |
| *BRAF*-del21-24 | tcctacATCCTATGAAAATGTTGAATGCTCATTACTCTTGAATAGAAGTGACCTA |
| *BRAF*-del25-28 | tcctacATCCTATGAAAATGTTGAATGCTCAGCAGTCTTGAATAGAAGTGACCTA |
| *BRAF*-in5-8 | tcctacAATGATCCTAAATGTTGAATGCTCAGCAGTTACTCTTGAATAGAAGTGACCTA |
| *BRAF*-in9-12 | tcctacAATGAAAATTCCTGTTGAATGCTCAGCAGTTACTCTTGAATAGAAGTGACCTA |
| *BRAF*-in13-16 | tcctacAATGAAAATGTTGTCCTAATGCTCAGCAGTTACTCTTGAATAGAAGTGACCTA |
| *BRAF*-in17-20 | tcctacAATGAAAATGTTGAATGTCCTCTCAGCAGTTACTCTTGAATAGAAGTGACCTA |
| *BRAF*-in21-24 | tcctacAATGAAAATGTTGAATGCTCATCCTGCAGTTACTCTTGAATAGAAGTGACCTA |
| *BRAF*-in25-28 | tcctacAATGAAAATGTTGAATGCTCAGCAGTCCTTTACTCTTGAATAGAAGTGACCTA |

* The T7 promoter sequence is labeled in green. Mismatch sites are highlighted in red. The forward and reverse primers serve as templates for each other during PCR amplification. The resulting products are then transcribed using T7 RNA polymerase, followed by RNA purification and quantification to obtain RNA templates containing mutations or indels.

Table S4 Sequence information for *in vitro* transcription of

*NPM1,* PA *and BRAF* LwaCas13a crRNA

| **Name** | ***Sequence (5′-3′)** |
| --- | --- |
| LwaCas13a-crRNA-FP | GAAATTAATACGACTCACTATAGGGGATTTAGACTACCCCAAAAACGAAGGGGACTAAA |
| LwaCas13a-crRNA 9-12F-RP | GATCTCTGCCTGGCAGTGGAGGAAGTCTGTTTTAGTCCCCTTCGTTTTTGGGGTAGTCT |
| LwaCas13a-crRNA 11-14F-RP | AAGATCTCTGCCTGGCAGTGGAGGAAGTGTTTTAGTCCCCTTCGTTTTTGGGGTAGTCT |
| LwaCas13a-crRNA 13-16F-RP | TCAAGATCTCTGCCTGGCAGTGGAGGAAGTTTTAGTCCCCTTCGTTTTTGGGGTAGTCT |
| LwaCas13a-crRNA 15-18F-RP | ATTCAAGATCTCTGCCTGGCAGTGGAGGGTTTTAGTCCCCTTCGTTTTTGGGGTAGTCT |
| LwaCas13a-crRNA 17-20F-RP | CTATTCAAGATCTCTGCCTGGCAGTGGAGTTTTAGTCCCCTTCGTTTTTGGGGTAGTCT |
| LwaCas13a-crRNA 9-12R-RP | AGACTTCCTCCACTGCCAGGCAGAGATCGTTTTAGTCCCCTTCGTTTTTGGGGTAGTCT |
| LwaCas13a-crRNA 11-14R-RP | CTTCCTCCACTGCCAGGCAGAGATCTTGGTTTTAGTCCCCTTCGTTTTTGGGGTAGTCT |
| LwaCas13a-crRNA 13-16R-RP | TTCCTCCACTGCCAGGCAGAGATCTTGAGTTTTAGTCCCCTTCGTTTTTGGGGTAGTCT |
| LwaCas13a-crRNA 15-18R-RP | CCTCCACTGCCAGGCAGAGATCTTGAATGTTTTAGTCCCCTTCGTTTTTGGGGTAGTCT |
| LwaCas13a-crRNA 17-20R-RP | TCCACTGCCAGGCAGAGATCTTGAATAGGTTTTAGTCCCCTTCGTTTTTGGGGTAGTCT |
| LwaCas13a-crRNA PA-RP | TCGAGGCGAACATCGTCTGGTAACGCACGTTTTAGTCCCCTTCGTTTTTGGGGTAGTCT |
| LwaCas13a-crRNA *BRAF*-RP | GTAACTGCTGAGCATTCAACATTTTCATGTTTTAGTCCCCTTCGTTTTTGGGGTAGTCT |
| ssRNA reporter | 5’FAM-UUUUUC-3’BHQ1 |

* The T7 promoter sequence is labeled in green. The underlined sequence serves as the template for generating the LwaCas13a crRNA spacer. The red font indicates the 4-bp insertion mutations.

Table S5 Oligonucleotide sequences used for the preparation of *NPM1*-WT

and *NPM1*-insCCTG template RNAs

| **Name** | ***Sequence (5′-3′)** |
| --- | --- |
| *NPM*1-ForT7-Fm2 | GAAATTAATACGACTCACTATAGGGTACAATCGCTTCTTTAGGTCACTTCTATTCAAGA |
| *NPM*1-For-Rm2 | TACCTATGTTCGCTGTTGGTATCGCACCAGCTACTCGATCTGTACACACAAGACTTCCT |
| *NPM*1-Rev-Fm2 | TACCTATGTTCGCTGTTGGTATCGCACCAGCTACTCGATTAGGTCACTTCTATTCAAGA |
| *NPM*1-RevT7-Rm2 | GAAATTAATACGACTCACTATAGGGTACAATCGCTTCTTCTGTACACACAAGACTTCCT |
| *NPM*1-WT2 | TTAGGTCACTTCTATTCAAGATCTCTGGCAGTGGAGGAAGTCTTGTGTGTACAGA |
| *NPM*1-CCTG2 | TTAGGTCACTTCTATTCAAGATCTCTGCCTGGCAGTGGAGGAAGTCTTGTGTGTACAGA |

* The T7 promoter sequence is labeled in green. The positions of insertions are highlighted in red font. The insertion bases are highlighted in blue font. The forward primer, reverse primer, and amplification template undergo a PCR amplification reaction. The resulting products are then transcribed using T7 RNA polymerase, followed by RNA purification and quantification to obtain RNA templates containing mutations or indels.

**Table S6 Oligonucleotide sequences for the preparation of RNA templates**

for 40 types of *NPM1* c.863_864 4-bp insertions

| **Name** | ***Sequence (5′-3′)** |
| --- | --- |
| *NPM1*-Rev-Fm2 | TACCTATGTTCGCTGTTGGTATCGCACCAGCTACTCGATTAGGTCACTTCTATTCAAGA |
| *NPM1*-RevT7-Rm2 | GAAATTAATACGACTCACTATAGGGTACAATCGCTTCTTCTGTACACACAAGACTTCCT |
| *NPM1*-WT2 | TTAGGTCACTTCTATTCAAGATCTCTGGCAGTGGAGGAAGTCTTGTGTGTACAGA |
| *NPM1*-1CCTC1 | TTAGGTCACTTCTATTCAAGATCTCTGCCTCGCAGTGGAGGAAGTCTTGTGTGTACAGA |
| *NPM1*-1ACTG2 | TTAGGTCACTTCTATTCAAGATCTCTGACTGGCAGTGGAGGAAGTCTTGTGTGTACAGA |
| *NPM1*-1GCTG3 | TTAGGTCACTTCTATTCAAGATCTCTGGCTGGCAGTGGAGGAAGTCTTGTGTGTACAGA |
| *NPM1*-1CGTG4 | TTAGGTCACTTCTATTCAAGATCTCTGCGTGGCAGTGGAGGAAGTCTTGTGTGTACAGA |
| *NPM1*-1CCCG5 | TTAGGTCACTTCTATTCAAGATCTCTGCCCGGCAGTGGAGGAAGTCTTGTGTGTACAGA |
| *NPM1*-1CCTA6 | TTAGGTCACTTCTATTCAAGATCTCTGCCTAGCAGTGGAGGAAGTCTTGTGTGTACAGA |
| *NPM1*-1TCTG7 | TTAGGTCACTTCTATTCAAGATCTCTGTCTGGCAGTGGAGGAAGTCTTGTGTGTACAGA |
| *NPM1*-1CATG8 | TTAGGTCACTTCTATTCAAGATCTCTGCATGGCAGTGGAGGAAGTCTTGTGTGTACAGA |
| *NPM1*-1CTTG9 | TTAGGTCACTTCTATTCAAGATCTCTGCTTGGCAGTGGAGGAAGTCTTGTGTGTACAGA |
| *NPM1*-1CCAG10 | TTAGGTCACTTCTATTCAAGATCTCTGCCAGGCAGTGGAGGAAGTCTTGTGTGTACAGA |
| *NPM1*-2CAAG11 | TTAGGTCACTTCTATTCAAGATCTCTGCAAGGCAGTGGAGGAAGTCTTGTGTGTACAGA |
| *NPM1*-2CACG12 | TTAGGTCACTTCTATTCAAGATCTCTGCACGGCAGTGGAGGAAGTCTTGTGTGTACAGA |
| *NPM1*-2CCAC13 | TTAGGTCACTTCTATTCAAGATCTCTGCCACGCAGTGGAGGAAGTCTTGTGTGTACAGA |
| *NPM1*-2CCGA14 | TTAGGTCACTTCTATTCAAGATCTCTGCCGAGCAGTGGAGGAAGTCTTGTGTGTACAGA |
| *NPM1*-2TGTG15 | TTAGGTCACTTCTATTCAAGATCTCTGTGTGGCAGTGGAGGAAGTCTTGTGTGTACAGA |
| *NPM1*-2TTTG16 | TTAGGTCACTTCTATTCAAGATCTCTGTTTGGCAGTGGAGGAAGTCTTGTGTGTACAGA |
| *NPM1*-2CCAA17 | TTAGGTCACTTCTATTCAAGATCTCTGCCAAGCAGTGGAGGAAGTCTTGTGTGTACAGA |
| *NPM1*-2CTAG18 | TTAGGTCACTTCTATTCAAGATCTCTGCTAGGCAGTGGAGGAAGTCTTGTGTGTACAGA |
| *NPM1*-2ACAG19 | TTAGGTCACTTCTATTCAAGATCTCTGACAGGCAGTGGAGGAAGTCTTGTGTGTACAGA |
| *NPM1*-2CATA20 | TTAGGTCACTTCTATTCAAGATCTCTGCATAGCAGTGGAGGAAGTCTTGTGTGTACAGA |
| *NPM1*-3CAAA21 | TTAGGTCACTTCTATTCAAGATCTCTGCAAAGCAGTGGAGGAAGTCTTGTGTGTACAGA |
| *NPM1*-3CACA22 | TTAGGTCACTTCTATTCAAGATCTCTGCACAGCAGTGGAGGAAGTCTTGTGTGTACAGA |
| *NPM1*-3CGCC23 | TTAGGTCACTTCTATTCAAGATCTCTGCGCCGCAGTGGAGGAAGTCTTGTGTGTACAGA |
| *NPM1*-3TACG24 | TTAGGTCACTTCTATTCAAGATCTCTGTACGGCAGTGGAGGAAGTCTTGTGTGTACAGA |
| *NPM1*-3TAGG25 | TTAGGTCACTTCTATTCAAGATCTCTGTAGGGCAGTGGAGGAAGTCTTGTGTGTACAGA |
| *NPM1*-3TCAT26 | TTAGGTCACTTCTATTCAAGATCTCTGTCATGCAGTGGAGGAAGTCTTGTGTGTACAGA |
| *NPM1*-3TGCG27 | TTAGGTCACTTCTATTCAAGATCTCTGTGCGGCAGTGGAGGAAGTCTTGTGTGTACAGA |
| *NPM1*-3TCGT28 | TTAGGTCACTTCTATTCAAGATCTCTGTCGTGCAGTGGAGGAAGTCTTGTGTGTACAGA |
| *NPM1*-3ATAG29 | TTAGGTCACTTCTATTCAAGATCTCTGATAGGCAGTGGAGGAAGTCTTGTGTGTACAGA |
| *NPM1*-3TCAC30 | TTAGGTCACTTCTATTCAAGATCTCTGTCACGCAGTGGAGGAAGTCTTGTGTGTACAGA |
| *NPM1*-4TACT31 | TTAGGTCACTTCTATTCAAGATCTCTG TACTGCAGTGGAGGAAGTCTTGTGTGTACAGA |
| *NPM1*-4ATAT32 | TTAGGTCACTTCTATTCAAGATCTCTG ATATGCAGTGGAGGAAGTCTTGTGTGTACAGA |
| *NPM1*-4TTAT33 | TTAGGTCACTTCTATTCAAGATCTCTGTTATGCAGTGGAGGAAGTCTTGTGTGTACAGA |
| *NPM1*-4GACA34 | TTAGGTCACTTCTATTCAAGATCTCTGGACAGCAGTGGAGGAAGTCTTGTGTGTACAGA |
| *NPM1*-4AACA35 | TTAGGTCACTTCTATTCAAGATCTCTGAACAGCAGTGGAGGAAGTCTTGTGTGTACAGA |
| *NPM1*-4TGAC36 | TTAGGTCACTTCTATTCAAGATCTCTGTGACGCAGTGGAGGAAGTCTTGTGTGTACAGA |
| *NPM1*-4GTCA37 | TTAGGTCACTTCTATTCAAGATCTCTGGTCAGCAGTGGAGGAAGTCTTGTGTGTACAGA |
| *NPM1*-4ATAA38 | TTAGGTCACTTCTATTCAAGATCTCTGATAAGCAGTGGAGGAAGTCTTGTGTGTACAGA |
| *NPM1*-4TTAA39 | TTAGGTCACTTCTATTCAAGATCTCTGTTAAGCAGTGGAGGAAGTCTTGTGTGTACAGA |
| *NPM1*-4ATCA40 | TTAGGTCACTTCTATTCAAGATCTCTGATCAGCAGTGGAGGAAGTCTTGTGTGTACAGA |

* The T7 promoter sequence is in green. The forward primer, reverse primer, and amplification template undergo a PCR amplification reaction. The resulting products are then transcribed using T7 RNA polymerase, followed by RNA purification and quantification to obtain RNA templates containing mutations or indels.

**Table S7 Oligonucleotide sequences for the preparation of RNA templates**

for the top 11 most frequent *NPM1* c.863_864 4-bp insertions

| **Name** | ***Sequence (5′-3′)** |
| --- | --- |
| *NPM1*-Rev-Fm2 | TACCTATGTTCGCTGTTGGTATCGCACCAGCTACTCGATTAGGTCACTTCTATTCAAGA |
| *NPM1*-RevT7-Rm2 | GAAATTAATACGACTCACTATAGGGTACAATCGCTTCTTCTGTACACACAAGACTTCCT |
| *NPM1*-WT2 | TTAGGTCACTTCTATTCAAGATCTCTGGCAGTGGAGGAAGTCTTGTGTGTACAGA |
| *NPM1*-TCTG1 | TTAGGTCACTTCTATTCAAGATCTCTGTCTGGCAGTGGAGGAAGTCTTGTGTGTACAGA |
| *NPM1*-CATG2 | TTAGGTCACTTCTATTCAAGATCTCTGCATGGCAGTGGAGGAAGTCTTGTGTGTACAGA |
| *NPM1*-CCTG3 | TTAGGTCACTTCTATTCAAGATCTCTGCCTGGCAGTGGAGGAAGTCTTGTGTGTACAGA |
| *NPM1*-CCAG4 | TTAGGTCACTTCTATTCAAGATCTCTGCCAGGCAGTGGAGGAAGTCTTGTGTGTACAGA |
| *NPM1*-CCGG5 | TTAGGTCACTTCTATTCAAGATCTCTGCCGGGCAGTGGAGGAAGTCTTGTGTGTACAGA |
| *NPM1*-CTTG6 | TTAGGTCACTTCTATTCAAGATCTCTGCTTGGCAGTGGAGGAAGTCTTGTGTGTACAGA |
| *NPM1*-TATG7 | TTAGGTCACTTCTATTCAAGATCTCTGTATGGCAGTGGAGGAAGTCTTGTGTGTACAGA |
| *NPM1*-TCGG8 | TTAGGTCACTTCTATTCAAGATCTCTGTCGGGCAGTGGAGGAAGTCTTGTGTGTACAGA |
| *NPM1*-TAAG9 | TTAGGTCACTTCTATTCAAGATCTCTGTAAGGCAGTGGAGGAAGTCTTGTGTGTACAGA |
| *NPM1*-CAGG10 | TTAGGTCACTTCTATTCAAGATCTCTGCAGGGCAGTGGAGGAAGTCTTGTGTGTACAGA |
| *NPM1*-CAGA11 | TTAGGTCACTTCTATTCAAGATCTCTGCAGAGCAGTGGAGGAAGTCTTGTGTGTACAGA |

* The T7 promoter sequence is in green. The positions of insertions are highlighted in red font. The insertion bases are highlighted in blue font. The forward primer, reverse primer, and amplification template undergo a PCR amplification reaction. The resulting products are then transcribed using T7 RNA polymerase, followed by RNA purification and quantification to obtain RNA templates containing mutations or indels

Table S8 RPA Primer sequences used in CRISPR-Scan system

| **Name** | ***Sequence (5′-3′)** |
| --- | --- |
| *NPM1*-RPA-F1 | CTTTATCTAGAGTTAACTCTCTGGTGGTAGAATG |
| *NPM1*-RPA-F2 | ATAGATGTTGAACTATGCAAAGAGACATTTAAT |
| *NPM1*-RPA-F3 | CCTTAACCACATTTCTTTTTTTTTTTTTCCAGGC |
| *NPM1*-RPA-F4 | AAAAATAGATGTTGAACTATGCAAAGAGAC |
| *NPM1*-RPA-F5 | GATGTCTATGAAGTGTTGTGGTTCCTTAACCAC |
| *NPM1*-RPA-R1 | GAAATTAATACGACTCACTATAGGGACACGGTAGGGAAAGTTCTCACTCTGCATTAT |
| *NPM1*-RPA-R2 | GAAATTAATACGACTCACTATAGGGAGGACAGCCAGATATCAACTGTTACAGAAATG |
| *NPM1*-RPA-R3 | GAAATTAATACGACTCACTATAGGGATATCAACTGTTACAGAAATGAAATAAGACGG |
| *NPM1*-RPA-R4 | GAAATTAATACGACTCACTATAGGGATCAACTGTTACAGAAATGAAATAAGACGG |
| *NPM1*-RPA-R5 | GAAATTAATACGACTCACTATAGGGACTATTTTCTTAAAGAGACTTCCTCCACTG |
| *GAPDH*-RPA-F | GAAATTAATACGACTCACTATAGGGTACACTGAGCACCAGGTGGTCTCCTCTGAC |
| *GAPDH*-RPA-R | ACATACCAGGAAATGAGCTTGACAAAGTGG |

* The T7 promoter sequence is in green.

Table S9 Primer sequences for FGS

| **Target** | **Name** | **Sequence (5′-3′)** |
| --- | --- | --- |
| *NPM1* gene | *NPM1*-FGS-F | AGTTAACTCTCTGGTGGTAGA |
|  | *NPM1*-FGS-R | TCAAACACGGTAGGGAAAGTT |

Table S10 Primer sequences for Next-generation sequencing

| **Name** | ***Sequence (5′-3′)** |
| --- | --- |
| *NPM1*-NGS-F | ACACTCTTTCCCTACACGACGCTCTTCCGATCTNNNNCTATGCAAAGAGACATTTAAT |
| *NPM1*-NGS-R | ACTGGAGTTCAGACGTGTGCTCTTCCGATCTNNNNACAGAAATGAAATAAGACGGA |
| P5-index1-F | AATGATACGGCGACCACCGAGATCTACACTGAACCTTACACTCTTTCCCTACACGAC |
| P5-index2-F | AATGATACGGCGACCACCGAGATCTACACTGCTAAGTACACTCTTTCCCTACACGAC |
| P5-index3-F | AATGATACGGCGACCACCGAGATCTACACTAAGACACACACTCTTTCCCTACACGAC |
| P5-index4-F | AATGATACGGCGACCACCGAGATCTACACTGTTCTCTACACTCTTTCCCTACACGAC |
| P5-index5-F | AATGATACGGCGACCACCGAGATCTACACCTAATCGAACACTCTTTCCCTACACGAC |
| P5-index6-F | AATGATACGGCGACCACCGAGATCTACACCTAGAACAACACTCTTTCCCTACACGAC |
| P5-index7-F | AATGATACGGCGACCACCGAGATCTACACTAAGTTCCACACTCTTTCCCTACACGAC |
| P5-index8-F | AATGATACGGCGACCACCGAGATCTACACTAGACCTAACACTCTTTCCCTACACGAC |
| P5-index37-F | AATGATACGGCGACCACCGAGATCTACACTATAGCCTACACTCTTTCCCTACACGAC |
| P5-index38-F | AATGATACGGCGACCACCGAGATCTACACATAGAGGCACACTCTTTCCCTACACGAC |
| P5-index39-F | AATGATACGGCGACCACCGAGATCTACACCCTATCCTACACTCTTTCCCTACACGAC |
| P5-index40-F | AATGATACGGCGACCACCGAGATCTACACGGCTCTGAACACTCTTTCCCTACACGAC |
| P5-index41-F | AATGATACGGCGACCACCGAGATCTACACAGGCGAAGACACTCTTTCCCTACACGAC |
| P5-index42-F | AATGATACGGCGACCACCGAGATCTACACTAATCTTAACACTCTTTCCCTACACGAC |
| P5-index43-F | AATGATACGGCGACCACCGAGATCTACACCAGGACGTACACTCTTTCCCTACACGAC |
| P5-index44-F | AATGATACGGCGACCACCGAGATCTACACGTACTGACACACTCTTTCCCTACACGAC |
| P7-adapter25-R | CAAGCAGAAGACGGCATACGAGATCGAGTAATGTGACTGGAGTTCAGACGTGTG |
| P7-adapter26-R | CAAGCAGAAGACGGCATACGAGATTCTCCGGAGTGACTGGAGTTCAGACGTGTG |
| P7-adapter27-R | CAAGCAGAAGACGGCATACGAGATAATGAGCGGTGACTGGAGTTCAGACGTGTG |
| P7-adapter28-R | CAAGCAGAAGACGGCATACGAGATGGAATCTCGTGACTGGAGTTCAGACGTGTG |
| P7-adapter29-R | CAAGCAGAAGACGGCATACGAGATTTCTGAATGTGACTGGAGTTCAGACGTGTG |
| P7-adapter30-R | CAAGCAGAAGACGGCATACGAGATACGAATTCGTGACTGGAGTTCAGACGTGTG |
| P7-adapter31-R | CAAGCAGAAGACGGCATACGAGATAGCTTCAGGTGACTGGAGTTCAGACGTGTG |
| P7-adapter32-R | CAAGCAGAAGACGGCATACGAGATGCGCATTAGTGACTGGAGTTCAGACGTGTG |
| P7-adapter33-R | CAAGCAGAAGACGGCATACGAGATCATAGCCGGTGACTGGAGTTCAGACGTGTG |
| P7-adapter34-R | CAAGCAGAAGACGGCATACGAGATTTCGCGGAGTGACTGGAGTTCAGACGTGTG |
| P7-adapter35-R | CAAGCAGAAGACGGCATACGAGATGCGCGAGAGTGACTGGAGTTCAGACGTGTG |
| P7-adapter36-R | CAAGCAGAAGACGGCATACGAGATCTATCGCTGTGACTGGAGTTCAGACGTGTG |
| P7-adapter37-R | CAAGCAGAAGACGGCATACGAGATGTCGTGATGTGACTGGAGTTCAGACGTGTG |
| P7-adapter38-R | CAAGCAGAAGACGGCATACGAGATACCACTGTGTGACTGGAGTTCAGACGTGTG |
| P7-adapter39-R | CAAGCAGAAGACGGCATACGAGATTGGATCTGGTGACTGGAGTTCAGACGTGTG |
| P7-adapter40-R | CAAGCAGAAGACGGCATACGAGATCCGTTTGTGTGACTGGAGTTCAGACGTGTG |
| P7-adapter41-R | CAAGCAGAAGACGGCATACGAGATTGCTGGGTGTGACTGGAGTTCAGACGTGTG |
| P7-adapter42-R | CAAGCAGAAGACGGCATACGAGATGAGGGGTTGTGACTGGAGTTCAGACGTGTG |
| P7-adapter43-R | CAAGCAGAAGACGGCATACGAGATAGGTTGGGGTGACTGGAGTTCAGACGTGTG |
| P7-adapter44-R | CAAGCAGAAGACGGCATACGAGATGTGTGGTGGTGACTGGAGTTCAGACGTGTG |
| P7-adapter45-R | CAAGCAGAAGACGGCATACGAGATTGGGTTTCGTGACTGGAGTTCAGACGTGTG |
| P7-adapter46-R | CAAGCAGAAGACGGCATACGAGATTGGTCACAGTGACTGGAGTTCAGACGTGTG |
| P7-adapter47-R | CAAGCAGAAGACGGCATACGAGATTTGACCCTGTGACTGGAGTTCAGACGTGTG |
| P7-adapter48-R | CAAGCAGAAGACGGCATACGAGATCCACTCCTGTGACTGGAGTTCAGACGTGTG |

* *NPM1*-NGS-F and *NPM1*-NGS-R are the primers for the first PCR of NGS. P5 and P7 are different barcoded primers.

**Table S11. NPM1 mutation VAF and bone marrow blast percentage during MRD monitoring**

| Patient | Time from onset (months) | NPM1 mutation and VAF* | Bone marrow blasts (%)* |
| --- | --- | --- | --- |
| Patient-MRD-1 | 0 | insTCTG, 32.9% | 84.5% |
|  | 2 | insTCTG, 0.0% | 0.3% |
|  | 3 | insTCTG, 0.0% | 1.6% |
|  | 4 | insTCTG, 0.0% | 1.6% |
|  | 6 | insTCTG, 0.0% | 1.1% |
|  | 12 | insTCTG, 0.5% | 2.1% |
|  | 13 | insTCTG, 1.1% | 2.7% |
|  | 14 | insTCTG, 16.5% | 41.0% |
| Patient-MRD-3 | 0 | insTCTG, 42.7% | 97.0% |
|  | 1 | insTCTG, 0.0% | 0.0% |
|  | 9 | insTCTG, 22.6% | 41.1% |
| Patient-MRD-4 | 0 | insTCTG, 28.6% | 43.3% |
|  | 1 | insTCTG, 0.0% | 0.0% |
|  | 15 | insTCTG, 18.6% | 29.0% |

*A value of 0% indicates no detectable NPM1 mutation or bone marrow blasts.

Table S12 Oligonucleotide sequences used for the preparation of *KIT*, *BRAF*, *EGFR*, *GAPDH* LwaCas13a crRNA and corresponding template RNAs

| **Name** | ***Sequence (5′-3′)** |
| --- | --- |
| LwaCas13a-crRNA-FP | GAAATTAATACGACTCACTATAGGGGATTTAGACTACCCCAAAAACGAAGGGGACTAAA |
| *KIT*-LwaCas13a-crRNARP | ATGTATGAAGTACAGGTTGTTGAGGAGAGTTTTAGTCCCCTTCGTTTTTGGGGTAGTCT |
| *BRAF*-LwaCas13a-crRNARP | GTAACTGCTGAGCATTCAACATTTTCATGTTTTAGTCCCCTTCGTTTTTGGGGTAGTCT |
| *EGFR*-LwaCas13a-crRNARP | TTTCGGAGATGTTTTGATAGCGACGGGTGTTTTAGTCCCCTTCGTTTTTGGGGTAGTCT |
| *GAPDH* -LwaCas13a-crRNARP | ACAGCGACACCCACTCCTCCACCTTTGAGTTTTAGTCCCCTTCGTTTTTGGGGTAGTCT |
| TargetKBE-T7-Fm | GAAATTAATACGACTCACTATAGGG TACAATCGCTTCTTTAGGTCACTTCTATTCAAGA |
| *KIT* WT-Rm | TTCTTTAGGTCACTTCTATTCAAGATCTCCTCAACAACCTTCCACTGTACTTCATACAT |
| *KIT* c.1669_1674del-Rm | TCTCCTCAACAACCTGTACTTCATACATTCTTGAATAGAAGTGACCTAAAGAAGCGATT |
| *KIT* c.1670_1675del-Rm | TCTCCTCAACAAACTGTACTTCATACATTCTTGAATAGAAGTGACCTAAAGAAGCGATT |
| *BRAF* WT-Rm | TGACAGCACCTACACCTCAGCAGTTACTCTTGAATAGAAGTGACCTAAAGAAGCGATTG |
| *BRAF* c.1459_1473del-Rm | ATGAAAATGTTGAATCCTCAGCAGTTACTCTTGAATAGAAGTGACCTAAAGAAGCGATT |
| *BRAF* c.1460_1474del-Rm | ATGAAAATGTTGAATGCTCAGCAGTTACTCTTGAATAGAAGTGACCTAAAGAAGCGATT |
| *EGFR* WT-Rm | CCCGTCGCTATCAAGGAATTAAGAGAAGCAACATCTCCGAAATCTTGAATAGAAGTGAC |
| *EGFR* c.2235_2249del-Rm | ACCCGTCGCTATCAAAACATCTCCGAAATCTTGAATAGAAGTGACCTAAAGAAGCGATT |
| *EGFR* c.2236_2250del-Rm | ACCCGTCGCTATCAAGACATCTCCGAAATCTTGAATAGAAGTGACCTAAAGAAGCGATT |
| *EGFR* c.2237_2251del-Rm | ACCCGTCGCTATCAAGGCATCTCCGAAATCTTGAATAGAAGTGACCTAAAGAAGCGATT |

* The T7 promoter sequence is labeled in green. The forward primer, reverse primer, and amplification template undergo a PCR amplification reaction. The resulting products are then transcribed using T7 RNA polymerase, followed by RNA purification and quantification to obtain RNA templates containing mutations or indels.

**Sequence information of plasmids**

***> NPM1*-WT gene (533-nt)**

AACATTGGCCATATGGGTCTCTGTTCTTTCTGTTGATTTCCTTAAGTAAATAATGCTAAACTATTAAATAATTATTAGTATATTGTTCACATTTTTATGACTGATTAAAGTGTTTGGAATTAAATTACATCTGAGTATAAATTTTCTTGGAGTCATATCTTTATCTAGAGTTAACTCTCTGGTGGTAGAATGAAAAATAGATGTTGAACTATGCAAAGAGACATTTAATTTATTGATGTCTATGAAGTGTTGTGGTTCCTTAACCACATTTCTTTTTTTTTTTTTCCAGGCTATTCAAGATCTCTGGCAGTGGAGGAAGTCTCTTTAAGAAAATAGTTTAAACAATTTGTTAAAAAATTTTCCGTCTTATTTCATTTCTGTAACAGTTGATATCTGGCTGTCCTTTTTATAATGCAGAGTGAGAACTTTCCCTACCGTGTTTGATAAATGTTGTCCAGGTTCTATTGCCAAGAATGTGTTGTCCAAAATGCCTGTTTAGTTTTTAAAGATGGAACTCCACCCTTTGCTTGGTT

**> *NPM1*-insCCTG gene (537-nt)**

AACATTGGCCATATGGGTCTCTGTTCTTTCTGTTGATTTCCTTAAGTAAATAATGCTAAACTATTAAATAATTATTAGTATATTGTTCACATTTTTATGACTGATTAAAGTGTTTGGAATTAAATTACATCTGAGTATAAATTTTCTTGGAGTCATATCTTTATCTAGAGTTAACTCTCTGGTGGTAGAATGAAAAATAGATGTTGAACTATGCAAAGAGACATTTAATTTATTGATGTCTATGAAGTGTTGTGGTTCCTTAACCACATTTCTTTTTTTTTTTTTCCAGGCTATTCAAGATCTCTGCCTGGCAGTGGAGGAAGTCTCTTTAAGAAAATAGTTTAAACAATTTGTTAAAAAATTTTCCGTCTTATTTCATTTCTGTAACAGTTGATATCTGGCTGTCCTTTTTATAATGCAGAGTGAGAACTTTCCCTACCGTGTTTGATAAATGTTGTCCAGGTTCTATTGCCAAGAATGTGTTGTCCAAAATGCCTGTTTAGTTTTTAAAGATGGAACTCCACCCTTTGCTTGGTT

**> *NPM1*-insTCTG gene (537-nt)**

AACATTGGCCATATGGGTCTCTGTTCTTTCTGTTGATTTCCTTAAGTAAATAATGCTAAACTATTAAATAATTATTAGTATATTGTTCACATTTTTATGACTGATTAAAGTGTTTGGAATTAAATTACATCTGAGTATAAATTTTCTTGGAGTCATATCTTTATCTAGAGTTAACTCTCTGGTGGTAGAATGAAAAATAGATGTTGAACTATGCAAAGAGACATTTAATTTATTGATGTCTATGAAGTGTTGTGGTTCCTTAACCACATTTCTTTTTTTTTTTTTCCAGGCTATTCAAGATCTCTGTCTGGCAGTGGAGGAAGTCTCTTTAAGAAAATAGTTTAAACAATTTGTTAAAAAATTTTCCGTCTTATTTCATTTCTGTAACAGTTGATATCTGGCTGTCCTTTTTATAATGCAGAGTGAGAACTTTCCCTACCGTGTTTGATAAATGTTGTCCAGGTTCTATTGCCAAGAATGTGTTGTCCAAAATGCCTGTTTAGTTTTTAAAGATGGAACTCCACCCTTTGCTTGGTT

**> *NPM1*-insCATG gene (537-nt)**

AACATTGGCCATATGGGTCTCTGTTCTTTCTGTTGATTTCCTTAAGTAAATAATGCTAAACTATTAAATAATTATTAGTATATTGTTCACATTTTTATGACTGATTAAAGTGTTTGGAATTAAATTACATCTGAGTATAAATTTTCTTGGAGTCATATCTTTATCTAGAGTTAACTCTCTGGTGGTAGAATGAAAAATAGATGTTGAACTATGCAAAGAGACATTTAATTTATTGATGTCTATGAAGTGTTGTGGTTCCTTAACCACATTTCTTTTTTTTTTTTTCCAGGCTATTCAAGATCTCTGCATGGCAGTGGAGGAAGTCTCTTTAAGAAAATAGTTTAAACAATTTGTTAAAAAATTTTCCGTCTTATTTCATTTCTGTAACAGTTGATATCTGGCTGTCCTTTTTATAATGCAGAGTGAGAACTTTCCCTACCGTGTTTGATAAATGTTGTCCAGGTTCTATTGCCAAGAATGTGTTGTCCAAAATGCCTGTTTAGTTTTTAAAGATGGAACTCCACCCTTTGCTTGGTT

**> *NPM1*-insTAAG gene (537-nt)**

AACATTGGCCATATGGGTCTCTGTTCTTTCTGTTGATTTCCTTAAGTAAATAATGCTAAACTATTAAATAATTATTAGTATATTGTTCACATTTTTATGACTGATTAAAGTGTTTGGAATTAAATTACATCTGAGTATAAATTTTCTTGGAGTCATATCTTTATCTAGAGTTAACTCTCTGGTGGTAGAATGAAAAATAGATGTTGAACTATGCAAAGAGACATTTAATTTATTGATGTCTATGAAGTGTTGTGGTTCCTTAACCACATTTCTTTTTTTTTTTTTCCAGGCTATTCAAGATCTCTGTAAGGCAGTGGAGGAAGTCTCTTTAAGAAAATAGTTTAAACAATTTGTTAAAAAATTTTCCGTCTTATTTCATTTCTGTAACAGTTGATATCTGGCTGTCCTTTTTATAATGCAGAGTGAGAACTTTCCCTACCGTGTTTGATAAATGTTGTCCAGGTTCTATTGCCAAGAATGTGTTGTCCAAAATGCCTGTTTAGTTTTTAAAGATGGAACTCCACCCTTTGCTTGGTT

**> *NPM1*-insCAGA gene (537-nt)**

AACATTGGCCATATGGGTCTCTGTTCTTTCTGTTGATTTCCTTAAGTAAATAATGCTAAACTATTAAATAATTATTAGTATATTGTTCACATTTTTATGACTGATTAAAGTGTTTGGAATTAAATTACATCTGAGTATAAATTTTCTTGGAGTCATATCTTTATCTAGAGTTAACTCTCTGGTGGTAGAATGAAAAATAGATGTTGAACTATGCAAAGAGACATTTAATTTATTGATGTCTATGAAGTGTTGTGGTTCCTTAACCACATTTCTTTTTTTTTTTTTCCAGGCTATTCAAGATCTCTGCAGAGCAGTGGAGGAAGTCTCTTTAAGAAAATAGTTTAAACAATTTGTTAAAAAATTTTCCGTCTTATTTCATTTCTGTAACAGTTGATATCTGGCTGTCCTTTTTATAATGCAGAGTGAGAACTTTCCCTACCGTGTTTGATAAATGTTGTCCAGGTTCTATTGCCAAGAATGTGTTGTCCAAAATGCCTGTTTAGTTTTTAAAGATGGAACTCCACCCTTTGCTTGGTT

The insertion sites are highlighted in yellow, and the inserted sequences are highlighted in green.
